# Targeted Genomic Sequencing of *TSC1* and *TSC2* Reveals Causal Variants in Individuals for Whom Previous Genetic Testing for Tuberous Sclerosis Complex Was Normal

**DOI:** 10.1155/2023/4899372

**Published:** 2023-07-13

**Authors:** Hannah D. West, Mark Nellist, Rutger W. W. Brouwer, Mirjam C. G. N. van den Hout-van Vroonhoven, Luiz Gustavo Dufner de Almeida, Femke Hendriks, Peter Elfferich, Meera Raja, Peter Giles, Rosa M. Alfano, Angela Peron, Yves Sznajer, Liesbeth De Waele, Anna Jansen, Marije Koopmans, Anneke Kievit, Laura S. Farach, Hope Northrup, Julian R. Sampson, Laura E. Thomas, Wilfred F. J. van IJcken

**Affiliations:** ^1^Institute of Medical Genetics, Division of Cancer and Genetics, Cardiff University, School of Medicine, Cardiff, UK; ^2^Department of Clinical Genetics, Erasmus Medical Center, Rotterdam, Netherlands; ^3^Center for Biomics and Department of Cell Biology, Erasmus Medical Center, Rotterdam, Netherlands; ^4^The Wales Gene Park, Division of Cancer and Genetics, Cardiff University, School of Medicine, Cardiff, UK; ^5^Medical Genetics, ASST Santi Paolo e Carlo, Ospedale San Paolo, Milan, Italy; ^6^Division of Medical Genetics, Department of Pediatrics, University of Utah School of Medicine, Salt Lake City, Utah, USA; ^7^Center for Human Genetics, Cliniques Universitaires Saint–Luc, UC Louvain, Brussels, Belgium; ^8^Department of Pediatrics and Child Neurology, University Hospital Leuven, Leuven, Belgium; ^9^Department of Pediatrics, Pediatric Neurology Unit, UZ Brussel, Brussels, Belgium; ^10^Department of Translational Neurosciences, University of Antwerp, Antwerp, Belgium; ^11^Department of Clinical Genetics, University Medical Center Utrecht, Utrecht, Netherlands; ^12^Department of Pediatrics, Division of Medical Genetics, McGovern Medical School at the University of Texas Health Science Center at Houston (UTHealth Houston) and Children's Memorial Hermann Hospital, Houston, Texas, USA; ^13^Institute of Life Science 1, Swansea University Medical School, Swansea, UK

## Abstract

Tuberous sclerosis complex (TSC) is caused by inactivating variants in *TSC1* and *TSC2*. Somatic mosaicism, as well as the size and complexity of the *TSC1* and *TSC2* loci, makes variant identification challenging. Indeed, in some individuals with a clinical diagnosis of TSC, diagnostic testing fails to identify an inactivating variant. To improve *TSC1* and *TSC2* variant detection, we screened the *TSC1* and *TSC2* genomic regions using targeted HaloPlex custom capture and next-generation sequencing (NGS) in genomic DNA isolated from peripheral blood of individuals with definite, possible or suspected TSC in whom no disease-associated variant had been identified by previous diagnostic genetic testing. We obtained >95% target region coverage at a read depth of 20 and >50% coverage at a read depth of 300 and identified inactivating *TSC1* or *TSC2* variants in 83/155 individuals (54%); 65/113 (58%) with clinically definite TSC and 18/42 (43%) with possible or suspected TSC. These included 19 individuals with deep intronic variants and 54 likely cases of mosaicism (variant allele frequency 1-28%; median 7%). In 13 cases (8%), we identified a variant of uncertain significance (VUS). Targeted genomic NGS of *TSC1* and *TSC2* increases the yield of inactivating variants found in individuals with suspected TSC.

## 1. Introduction

Tuberous sclerosis complex (TSC) is an autosomal dominant condition characterised by seizures, neuropsychiatric disorders, and the development of hamartomas in the brain, lungs, heart, skin and kidneys [1]. Loss-of-function variants in the TSC complex subunit 1 (*TSC1*; chromosome 9q34; OMIM 605284) or TSC complex subunit 2 (*TSC2*; chromosome 16p13.3; OMIM 191092) tumour suppressor genes cause TSC [1]. *TSC1* consists of 23 exons that extend across 60 kb of genomic DNA and produce an 8.5 kb mRNA encoding the 130 kDa TSC1 protein. The 46 kb *TSC2* locus consists of 42 exons that produce a 5.5 kb mRNA encoding the 200 kDa TSC2 protein. TSC1 and TSC2 interact to form the TSC complex, a negative regulator of the mechanistic target of rapamycin (mTOR) complex 1 (TORC1). Signal transduction through TORC1 controls key aspects of metabolism [2] and constitutive TORC1 activation is a hallmark of TSC-associated lesions.

The manifestations of TSC and their severity vary widely, and the identification of an inactivating *TSC1* or *TSC2* variant can help establish a diagnosis and enable cascade, preimplantation and prenatal genetic testing [3]. Some disease-associated *TSC1* and *TSC2* variants are found in multiple, unrelated individuals with TSC, but often, a unique variant is identified, and in most cases, the identified variant is the result of a *de novo* mutation [4, 5], either in a gamete or during (early) post-zygotic development [6–8]. The *TSC1* and *TSC2* Leiden Open Variation Databases (LOVD; http://www.lovd.nl/TSC1 and http://www.lovd.nl/TSC2) list many of the variants identified to date, alongside reports of predicted pathogenicity and functional test results. The wide variety of mutation types, ranging from single nucleotide changes to extensive chromosomal rearrangements, combined with the size and complexity of the *TSC1* and *TSC2* loci and the occurrence of mosaicism, makes the comprehensive identification of variants that cause TSC challenging. Indeed, in 10-15% of individuals with a clinically definite diagnosis of TSC, no causal variant is detected [4, 6–8]. These individuals are usually referred to as TSC “no mutation identified” (NMI). The failure to identify a causal variant can be due to technical issues associated with the screening method(s) employed or because the variant is located outside the screened region. Next-generation sequencing (NGS) has proven to be effective at overcoming some of these limitations [5, 6], and both whole exome sequencing (WES) and whole genome sequencing (WGS) are increasingly being applied as first-line diagnostic tests to identify individuals with TSC [5]. However, WES is not able to detect variants located deep within intronic sequences, and neither WES nor WGS is optimized for the efficient detection of post-zygotic mutations.

HaloPlex custom capture NGS relies on the specific capture of both ends of restriction-digested genomic DNA fragments from a region of interest, simplifying data analysis [9]. Previously, we showed in a small cohort of 6 TSC NMI individuals that HaloPlex custom capture could identify post-zygotic and deep intronic variants [10]. Here, we apply the same approach to a much larger TSC NMI cohort. Our data show that HaloPlex custom capture is an effective approach for the identification of otherwise difficult-to-detect *TSC1* and *TSC2* variants, particularly post-zygotic mutations. Where possible, we confirmed the HaloPlex results with a complementary DNA-based test and performed functional experiments to obtain evidence for pathogenicity at the mRNA or protein level. Our findings support the utility of bespoke NGS-based genetic analysis for variant detection in TSC and demonstrate the importance of functional approaches towards helping determine variant pathogenicity.

## 2. Methods

### 2.1. Editorial Policies and Ethical Considerations

Informed consent was provided by all subjects. All individuals had requested genetic testing of *TSC1* and *TSC2* for diagnostic purposes, and informed consent was provided as required by the institutional review board of the Erasmus Medical Center (EMC)(METC-2012-387), the NHS research ethics committee for Wales (REC 11WA0276), and the referring institution, according to standard diagnostic protocols.

### 2.2. Patient Cohort

Subjects had been referred for testing to the EMC, Rotterdam, Netherlands, or the Institute of Medical Genetics, Cardiff, UK, because of a diagnosis of definite or possible TSC [3], or who were suspected of TSC but had inadequate clinical details for classification, and were TSC NMI after diagnostic testing that included analysis of all coding exons and intron-exon boundaries by PCR and Sanger sequencing approaches, and multiplex ligation probe amplification (MLPA) for detection of large rearrangements.

### 2.3. DNA and RNA Isolation

Genomic DNA and total RNA were extracted from peripheral blood, affected and normal skin samples, and/or cultured skin fibroblasts using standard procedures. DNA quality and concentration were checked with the Quant-iT PicoGreen dsDNA Kit (Invitrogen, Carlsbad, USA).

### 2.4. HaloPlex Custom Capture NGS

Genomic DNA samples were subjected to customised HaloPlex or HaloPlex HS target enrichment assays (Agilent Technologies, Santa Clara, USA) encompassing the *TSC1* and *TSC2* genomic loci [9, 10]. See Supplementary Information, Methods for details.

### 2.5. Bioinformatics Analysis

Bioinformatic analysis was performed as described previously [10, 11]. See Supplementary Information, Methods for details. Reads were mapped to reference sequences NG_012386.1 (TSC1) and NG_005895.1 (TSC2) of build GRCh37 (hg19) of the human genome, and variants were annotated according to reference transcripts NM_000368.4 (TSC1) and NM_000548.3 (TSC2) unless specified otherwise.

### 2.6. Validation of Identified Variants

Likely germline changes were validated using a combination of PCR and Sanger sequencing. Post-zygotic changes were validated by allele-specific (AS) PCR, droplet digital (DD) PCR, or Nextera XT NGS. See Supplementary Methods for details.

To investigate effects on pre-mRNA splicing, RNA was isolated from blood or cultured skin fibroblasts, converted to cDNA using a cDNA synthesis kit (PCR Biosystems), and amplified by PCR. PCR products were analysed by agarose gel electrophoresis and Sanger sequencing. In some cases where no RNA was available, effects on pre-mRNA splicing were investigated using an *in vitro* exon trapping approach, as described previously [12]. See Supplementary Information, Methods, and Supplementary Tables S7 and S8 for details. Transcriptome sequencing was performed as described previously [13].

The effects of missense and in-frame deletion variants on the TSC complex and on TORC1 activity were assessed *in vitro*, as described previously [12].

Large deletions, affecting multiple exons, were validated either by MLPA (MRC Holland, Amsterdam, Netherlands) or using the GSA-MD-24 global screening single nucleotide polymorphism (SNP) array (Illumina).

## 3. Results

### 3.1. TSC NMI Cohort Characteristics

The cohort consisted of 155 TSC NMI individuals. According to the current clinical criteria [3], 113 (73%) had definite TSC, 34 (22%) had possible TSC, and 8 (5%) were suspected of TSC, but details of their clinical findings were not available to us. The clinical findings are summarised in the Supplementary Information, Tables S4–S6. In addition to testing single individuals, we tested 2 affected duos, 7 duos consisting of an affected subject plus an unaffected first-degree relative, and 38 trios consisting of an affected subject and both unaffected parents. In 6 cases, multiple genomic DNA samples from different tissues of a single individual were analysed.

### 3.2. *TSC1* and *TSC2* Variant Identification

We used 5 different HaloPlex custom capture designs, as detailed in the Supplementary Information, Methods, and Table S1. For each design, we obtained an average of >95% coverage of both target regions at a minimum depth of 20 reads per nucleotide, >85% coverage at a depth of 100 reads, and >50% coverage at a read depth of 300 (Supplementary Information, Table S2; the median read depth and range per subject sample is provided in Supplementary Information, Table S3).

**Figure 1:**
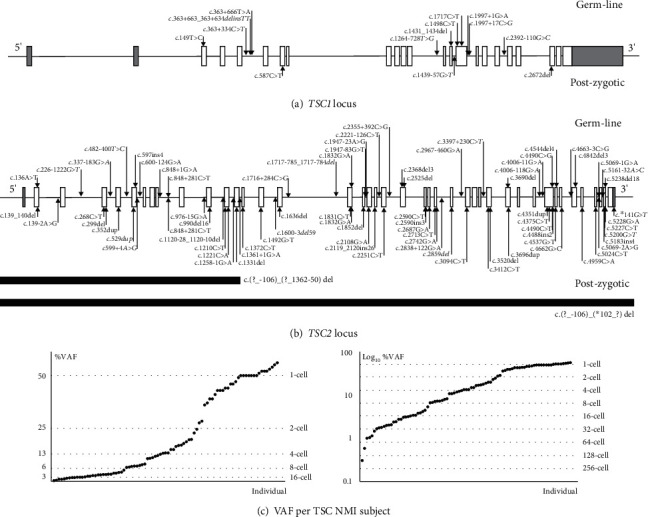
Overview of *TSC1* and *TSC2* variants identified using HaloPlex custom capture. The coding (open boxes) and noncoding exons (shaded grey) of both genes are shown, and the approximate positions of the identified variants indicated with arrows, except for the 2 large *TSC2* deletions which are shown as black bars. Germline variants (see Table 1) are shown above the corresponding gene; post-zygotic variants (see Table 2) are below. Variants of uncertain clinical significance and unconfirmed variants (see Table 3) are shown in italics. (a) Variants identified at the *TSC1* locus. (b) Variants identified at the *TSC2* locus. (c) Comparison of the variant allele frequencies (VAF) of the variants shown in (a) and (b) per TSC NMI subject. Subjects (*x*-axis) were ranked according to the percentage VAF (%VAF) and plotted according to a normal (left) and logarithmic scale (right). Dotted lines indicate the expected VAF for variants arising during the initial embryonic cell divisions. NMI: no mutation identified.

**Figure 2:**
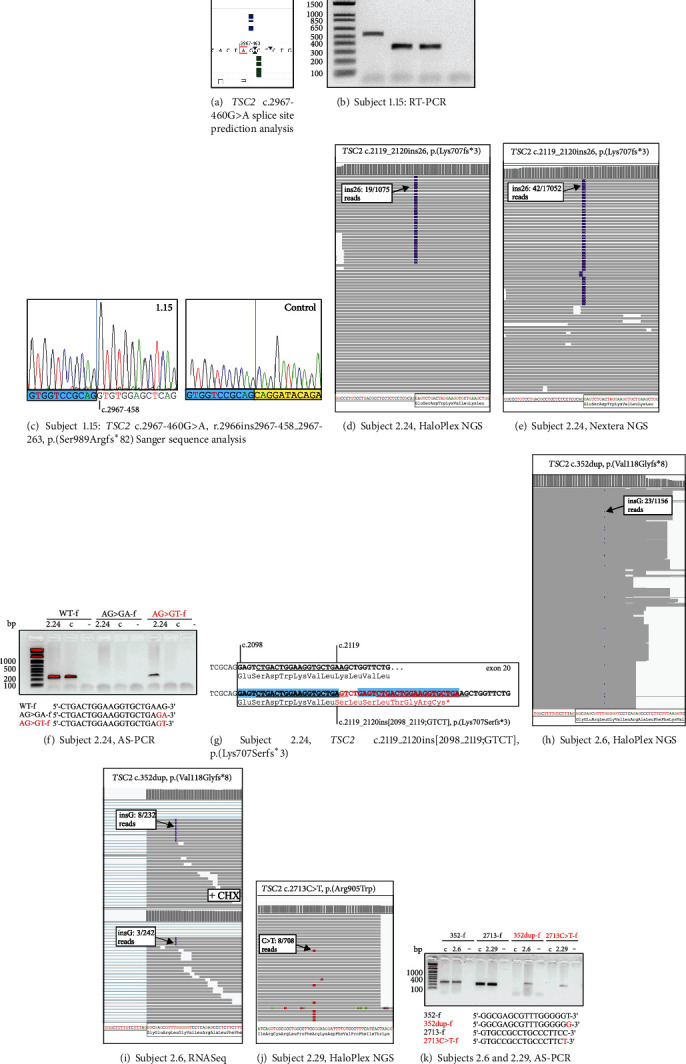
Examples of HaloPlex discovery and validation data for *TSC2* variants identified in the TSC ' no mutation identified' (NMI) cohort. (a–c) Subject 1.15, germline *TSC2* c.2967-460*G*>A variant. (a) Effect of the *TSC2* c.2967-460G>A variant on pre-mRNA splicing as predicted using the ALAMUT Visual Plus software package (version 1.7). Green blocks indicate a possible 3′ acceptor site, and blue blocks indicate a non-canonical 5′ donor site. (b) RT-PCR was performed on RNA isolated from subject 1.15, 2 control individuals (c), and a sample lacking RNA (-) using primers specific for *TSC2* exons 26 and 27. An abnormal product only was amplified from RNA from subject 1.15, most likely due to preferential amplification of the abnormal transcript. Exon 26 is skipped in the majority of wild-type *TSC2* transcripts in blood and the wild-type NM_000548.3(TSC2) transcript that includes exon 26 is often present at very low levels (data not shown). Size markers are indicated; bp, base pairs. (c) The Sanger sequencing of the RT-PCR products revealed the insertion of intronic sequence *TSC2* r.2966ins2967-458_2967-263, p.(Ser989Argfs^∗^82) in subject 1.15, but not in controls. Sequence corresponding to *TSC2* exons 26 and 27 is indicated in blue and yellow, respectively. (d–g) Subject 2.24, post-zygotic *TSC2* c.2119_2120ins[2098_2119;GTCT] variant. (d) Screenshot of the HaloPlex variant discovery data in the IGV. Reads are shown as grey bars; the insertion is shown in purple in multiple reads. The *TSC2* locus reference sequence is indicated; nucleotides corresponding to *TSC2* exon 20 are boxed. (e) Screenshot of the Nextera XT variant validation data in the IGV. Reads are shown as grey bars; the insertion is shown in purple in multiple reads. The *TSC2* locus reference sequence is shown in (d). (f) Allele-specific (AS)-PCR to show the presence of the *TSC2* c.2119_2120ins[2098_2119;GTCT] variant in genomic DNA from subject 2.24, and the absence of the variant from control samples with (c) or without (-) genomic DNA. Size markers are indicated; bp, base pairs. The AS primers are shown, with the variant-specific primer (AG>GT-f) and nucleotides indicated in red. (g) Schematic of the *TSC2* c.2119_2120ins[2098_2119;GTCT] variant. Nucleotides corresponding to the WT-f and AG>GT-f primers are underlined, the insertion is shown in red with the duplicated sequence shaded in blue. Sequences corresponding to *TSC2* exon 20 are boxed. (h–j) Subject 2.6, post-zygotic *TSC2* c.352dup variant, and subject 2.29, post-zygotic *TSC2* c.2713C>T variant. (h) Screenshot of the HaloPlex variant discovery data in the IGV for subject 2.6. Reads are shown as grey bars; a G insertion is shown in purple in multiple reads. The *TSC2* locus reference sequence is indicated; nucleotides corresponding to *TSC2* exon 5 are boxed. (i) Screenshot of RNASeq variant validation data in the IGV. Reads are shown as grey bars; the G insertion is shown in purple in multiple reads. The *TSC2* locus reference sequence is shown as in (h); RNA for RNASeq analysis was prepared from cultured skin fibroblasts; +CHX indicates that the fibroblasts were treated with cycloheximide. (j) Screenshot of the HaloPlex variant discovery data in the IGV for subject 2.29. Reads are shown as grey bars; a C>T transition is shown in red in multiple reads. The *TSC2* locus reference sequence is indicated; nucleotides corresponding to *TSC2* exon 24 are boxed. (k) AS-PCR to confirm the presence of the *TSC2* c.352dup and *TSC2* c.2713C>T variants in genomic DNA from subjects 2.6 and 2.29 respectively, but not in control genomic DNA samples (c) or in the absence of DNA (-). Size markers are indicated; bp, base pairs. The AS primers are shown, with the variant-specific primer and nucleotides indicated in red.

First, we searched for likely germline, inactivating *TSC1* and *TSC2* variants. We defined a minimum threshold of 50 reads (total) and a variant allele frequency (VAF) >40%, in line with a previous study [6]. In 2 affected individuals, from a 4-generation family with TSC, an obligate germline variant was identified with a VAF <40%, most likely due to reduced capture of restriction fragments containing the variant (Table 1; and see Supplemental Information, Figures S1 and S4). We identified from 0 to >70 germline variants per locus per individual, mostly known benign single nucleotide variants (SNVs), often present in multiple individuals in our cohort. Variants were classified according to the criteria of the American College of Medical Genetics and Genomics (ACMG) [14] and following recommendations from the *TSC1* and *TSC2* LOVD (http://www.lovd.nl/TSC1 and http://www.lovd.nl/TSC2). We identified a (likely) inactivating germline variant in 29 individuals: 7 in *TSC1* and 22 in *TSC2* (Table 1, Figure 1). In each case, we confirmed the presence of the variant by (i) visual inspection of the reads in the Integrated Genome Viewer (IGV) (http://www.broadinstitute.org/igv/) and (ii) PCR of genomic DNA from the corresponding individual, followed by the Sanger sequencing. To support the pathogenicity of variants predicted to affect TSC complex function or pre-mRNA splicing, functional testing (2 cases) or analysis of subject RNA (5 cases) was performed (Table 1; Figure 2; and see Supplementary Information, Figure S2).

Next, to identify post-zygotic *TSC1* and *TSC2* mutations, we searched for variants with a VAF <40%. Candidate (likely) causal variants were confirmed by visual inspection in the IGV and by either AS-PCR, DD-PCR, or Nextera XT NGS analysis of genomic DNA from the corresponding individual, together with appropriate controls (Table 2; Figure 2). Additional support for variant pathogenicity was sought, either by *in vitro* functional assessment of TSC complex activity (2 cases; see Supplementary Information, Figure S2), analysis of subject RNA (6 cases), or by *in vitro* exon trap experiments (6 cases; see Supplementary Information, Tables S7 and S8). To identify deletions >150 base pairs (bp) and other rearrangements that prevented fragment capture, we compared VAFs for SNVs across both loci and compared read depths using a *z*-score analysis [15]. We identified 2 post-zygotic *TSC2* deletions: subjects 2.52 and 2.53, estimated VAF: 15% and 10%, respectively. Both events were confirmed by MLPA or SNP array analysis (Table 2; and see Supplementary Information, Figure S3 and Table S9). In total, 54 (likely) inactivating post-zygotic variants were identified, 1 in *TSC1* and 53 in *TSC2*, accounting for 35% of the cohort (Table 2; Figure 1). In 5 individuals with an apparent inactivating postzygotic variant, we did not (yet) confirm the variant using a second test (Table 3), and in 13 individuals, we identified variants of uncertain significance (VUS) (Table 3; Figure 1).

### 3.3. Individuals with Multiple Genomic DNA Samples

In 6 cases, genomic DNA samples from different tissues from a single individual were tested.

In subject 1.14, a *TSC2* c.2525del p.(Pro842Hisfs∗52) variant was identified in genomic DNA isolated from a subependymal giant cell astrocytoma (SEGA) (VAF 51%) as well as from peripheral blood (VAF 48%) (Table 1).

In subject 3.7 with a SEGA but no other signs of TSC, a *TSC2* c.4375C>T, p.(Arg1459∗) variant was identified in the SEGA DNA (VAF 53%) but was absent from peripheral blood DNA (Table 3).

A *TSC2* c.5024C>T, p.(Pro1675Leu) variant (VAF 2%) was identified in genomic DNA isolated from a shagreen patch that was the only clinical sign of TSC in subject 3.19, but not in genomic DNA isolated from peripheral blood or from fibroblasts cultured from a biopsy of normal skin, either by HaloPlex NGS or by AS-PCR. This variant is likely a somatic event, specific to the shagreen patch (Table 3).

The *TSC2* c.5024C>T, p.(Pro1675Leu) variant was identified in genomic DNA samples isolated from both peripheral blood (VAF 24%) and normal skin fibroblasts (VAF 18%) from subject 2.46 (Table 2).

In subject 3.20 a novel variant in the overlapping 3′ UTR of *TSC2* and the polycystin 1, transient receptor potential channel interacting gene (*PKD1*) was detected in genomic DNA samples from peripheral blood and from an angiofibroma: *TSC2* c.∗141G>T, p.?; NM_001009944.2(PKD1):c.∗976C>, p.?; chr16:2138752G>T (Table 3). This variant might represent a first-hit event, but it is not clear how the variant inactivates *TSC2* and/or *PKD1*. Subject 3.20 did not have severe, early-onset renal cystic disease typically seen in individuals with inactivation of both genes [1] (see Supplementary Information, Table S6). An inactivating *TSC2* c.1331del, p.(Asn444Thrfs∗5) variant (VAF 3%) was identified in genomic DNA isolated from the angiofibroma but was absent from genomic DNA isolated from blood (Table 3) and is, therefore, likely to be a lesion-specific, second-hit mutation.

In subject 3.5 with definite TSC, a *TSC2* c.599+4A>G variant was detected in genomic DNA isolated from formalin-fixed paraffin-embedded (FFPE) SEGA tissue (VAF 30%), but not in genomic DNA isolated from peripheral blood. We failed to confirm the presence of the variant in the SEGA DNA, either by standard PCR followed by the Sanger sequencing, or by AS-PCR. Therefore, this individual remained NMI (Table 3).

### 3.4. Cases with Genomic DNA Samples from Multiple Family Members

We analysed 9 duos and 38 trios (see Tables 1–3). In 6 cases, a likely *de novo* germline variant was identified (Table 1). In 2 cases, the variant cosegregated with TSC: subjects 1.10 and 1.11 (Table 1) were both from a 4-generation family with TSC (see Supplementary Information, Figure S4) and subject 1.5 (Table 1) inherited an inactivating variant from subject 2.7 (Table 2), who was mosaic for the variant. In 16 cases, an affected child of healthy parents was mosaic for a *TSC2* variant (Table 2). In the remaining cases with multiple family members, no inactivating *TSC1* or *TSC2* variant was identified (see Supplementary Information, Table S6).

## 4. Discussion

We investigated a cohort of 155 individuals with a clinical diagnosis of definite or possible TSC, or with suspected TSC but with inadequate clinical details for classification, in whom previous genetic testing had not identified a causal variant. We identified an inactivating *TSC1* or *TSC2* variant in 83 (54%), including 65/113 (58%) of those with clinically definite TSC and 18/42 (43%) with possible TSC, or suspected of TSC but without sufficient clinical information for classification (Tables 1 and 2; and Supplementary Information Tables S4 and S5). In 4 cases, we identified an inactivating variant in genomic DNA isolated from affected tissue, but not in genomic DNA isolated from peripheral blood (Table 3). These most likely represent lesion-specific and/or second-hit events. In 13 cases (8%), we identified a variant but did not obtain sufficient evidence to establish or exclude pathogenicity (Table 3). Identification of an inactivating variant provided diagnostic certainty for the 18 individuals in whom TSC was suspected or could be defined only as “possible,” and in 83 cases, it provides the potential for prenatal or preimplantation genetic diagnostics and cascade testing for other family members, which was previously not possible.

Similar to a previous study of TSC NMI cases [6], 19/29 (66%) of the identified inactivating germline variants were located within sequences that had been screened during previous diagnostic testing, suggesting that simple technical issues account for a proportion of apparent TSC NMI cases. For example, we identified benign SNVs in *cis* that could have interfered with PCR primer binding (data not shown). In contrast, variants located deep within introns that interfere with *TSC1* or *TSC2* pre-mRNA splicing will never be identified by exon- or exome-based approaches. In 19 cases, we identified deep intronic variants (>10 nucleotides up or downstream from an exon), accounting for 12% of the cohort and 16/113 (14%) of the NMI cases with a clinical diagnosis of definite TSC. Evidence for or against variant pathogenicity was obtained either by family studies, analysis of RNA, or by *in vitro* exon trap experiments (Tables 1–3; see Supplementary Information, Tables S7 and S8). Notably, 2 recurrent deep intronic variants, *TSC2* c.2838-122G>A and *TSC2* c.848+281C>T, were identified in 10 unrelated cases, accounting for 6% of the cohort. We had originally identified the *TSC2* c.2838-122G>A variant in another individual [10] and have subsequently identified 2 further unrelated cases after targeted testing in our diagnostic laboratories (data not shown). The *TSC2* c.848+281C>T variant was reported previously in a separate study [6].

We identified an apparent post-zygotic mutation (VAF <40%) in 54 individuals (35% of the cohort), consistent with earlier reports of frequent mosaicism in TSC [6–8, 16] (Figure 1(c)). Detection of low-level mosaicism requires high-quality reads, deep coverage, and careful analysis of the data and is, therefore, easy to miss using routine diagnostic applications of WES or WGS [17]. The depth of coverage and the quality of the sequence reads following HaloPlex capture were variable and, in contrast to other studies [7, 16], we could not reliably detect variants with VAF <1%. Coverage at read depths >1000 was limited (Supplementary Information, Tables S1–S3), and although we did not observe a strong correlation between the median read depth per sample and the identification of a variant (Supplementary Information, Figure S5), it is likely that some low-frequency variants escaped detection. In mosaic individuals, the VAF may vary considerably between tissues, and testing multiple tissues, including hamartoma in which at least a proportion of cells should contain the first post-zygotic mutation, has been shown to be a fruitful approach [6–8, 16] and could also help resolve some of the additional remaining NMI cases in our cohort. Nonetheless, we identified and confirmed post-zygotic variants in genomic DNA from a significant proportion of the subjects (Table 2).

In addition to the limitations discussed above, there are 2 other reasons for our failure to detect a causal variant in all cases. First, some individuals who were tested might not have TSC (see Supplementary Information, Table S6). Second, the HaloPlex method is not able to efficiently capture junction fragments created by DNA rearrangements affecting >150 bp and is, therefore, not suited to detection of the large deletions and rearrangements that account for 3% (214/8202; search 1/6/2022) of the pathogenic *TSC2* variants and 0.5% (16/2964; search 1/6/2022) of the pathogenic *TSC1* variants listed in the *TSC2* and *TSC1* LOVD. We only identified 2 large post-zygotic *TSC2* deletions, accounting for <2% of our cohort (Table 2; and Supplementary Information, Figure S3), and failed to identify a known inversion at the *TSC2* locus in a control sample (data not shown).

Despite these caveats, our work shows the benefit of detailed analysis of the *TSC1* and *TSC2* genomic loci for TSC molecular diagnostics and indicates that targeted genomic NGS with high-quality reads and high read depth is an appropriate molecular screening method for individuals where there is a clinical suspicion of TSC, allowing reliable detection of both deep intronic variants that affect pre-mRNA splicing and low-frequency post-zygotic changes. The implementation of similar approaches in diagnostic laboratories could circumvent the requirements for either labour-intensive PCR-based exon-specific screening or inefficient WES/WGS approaches. However, the low number of cases identified with a VAF <1%, or with a large DNA rearrangement, suggests that other high read-depth approaches, particularly of genomic DNA isolated from multiple affected tissues [6–8, 16], might help solve more TSC NMI cases. Finally, our work has increased the spectrum of inactivating *TSC1* and *TSC2* variants associated with TSC and provides insight into the mechanisms of TSC pathogenesis.

## Figures and Tables

**Table 1:** Inactivating, likely germ-line *TSC1* and *TSC2* variants identified using HaloPlex custom capture NGS. Individuals fulfilling the clinical criteria for definite TSC [3] are indicated with “TSC”; those fulfilling only criteria for possible TSC are indicated with “?”; individuals for whom clinical information was not available to us are indicated with “n/a.” VAF, variant allele frequency, refers to the proportion of reads containing the corresponding variant. Cases for which multiple family members or multiple DNA samples were tested are indicated. Evidence for effects on pre-mRNA splicing was obtained by analysis of subject RNA isolated from either peripheral blood (RNA^1.^) or cultured skin fibroblasts (RNA^2.^). For functional studies please refer to the *TSC1* and *TSC2* Leiden Open Variation Databases (LOVD)(www.lovd.nl/TSC1, www.lovd.nl/TSC2). ClinVar (pathogenic, unless stated otherwise), the LOVD, and gnomAD, were accessed on 13/2/2023. P: pathogenic; LP: likely pathogenic; VUS: variant of uncertain clinical significance; LB: likely benign. Variants were classified according to the American College of Medical Genetics and Genomics (ACMG) criteria [15] using the ALAMUT Visual Plus (version 1.7) software package.

Subject	Clinical diagnosis	Variant hg19 (GRCh37); NG_005895.1, NM_000548.3(TSC2); NG_012386.1, NM_000368.4(TSC1)	VAF (%)	Evidence for pathogenicity	LOVD	ACMG criteria (classification)
1.1	?	*TSC2* c.136A>T, p.(Arg46^∗^), chr16:2098752A>T	122/302 (40%)	Stopgain; 1 × ClinVar: no assertion provided	P	PVS1, PM2, PP5 (LP)
1.2	n/a	*TSC2* c.597_598insTCGT, p.(Gln200Serfs^∗^36), chr16:2105518_2105519insTCGT	198/482 (41%)	Frameshift	novel	PVS1, PM2 (LP)
1.3, trio	TSC	*TSC2* c.600-124G>A, p.?, chr16:2106073G>A	797/1688 (47%)	*De novo,* strengthens cryptic donor site	novel	PM2,PP5, PS2 (VUS)
1.4	TSC	*TSC2* c.848+1G>A, p.?, chr16:2107180G>A	50/100 (50%)	Destroys canonical donor site; 3 × ClinVar	P	PM2, PP5 (VUS)
1.5, duo	TSC	*TSC2* c.848+281C>T, p.? chr16:2107460C>T	772/1554 (50%)	Creates a cryptic donor site; 3 × ClinVar; affected parent mosaic for the variant (see Table 2; subject 2.7)	P	PM2, PP5, PP1 (VUS)
1.6	?	*TSC2* c.848+281C>T, p.? chr16:2107460C>T	268/556 (48%)	Creates a cryptic donor site; 3 × ClinVar	P	PM2, PP5 (VUS)
1.7	TSC	*TSC2* c.1832G>A, p.(Arg611Gln), chr16:2120572G>A	368/726 (51%)	Missense; functional study (LOVD); 6 × ClinVar	P	PM1, PM2, PM5, PP3, PP5 (LP)
1.8	TSC	*TSC2* c.1832G>A, p.(Arg611Gln), chr16:2120572G>A	960/1786 (54%)	Missense; functional study (LOVD); 6 × ClinVar	P	PM1, PM2, PM5, PP3, PP5 (LP)
1.9	TSC	*TSC2* c.1947-83G>T, p.?, chr16:2121702G>T	941/2072 (45%)	Creates cryptic donor site	novel	PM2 (VUS)
1.10, duo	TSC	*TSC2* c.1947-23A>G, r.1947_2002del, p.(Glu650Alafs^∗^34), chr16:2121762A>G	175/538 (33%)	Disrupts acceptor site; RNA^2.^; co-segregation; affected parent of subject 1.11 (4 generation family, see Supplementary Information, Figure S4)	P	PM2, PP1, PP5 (VUS)
1.11, duo	n/a	*TSC2* c.1947-23A>G, r.1947_2002del, p.(Glu650Alafs^∗^34), chr16:2121762A>G	155/400 (39%)	Disrupts acceptor site; RNA^2.^; co-segregation; affected child of subject 1.10 (4 generation family, see Supplementary Information, Figure S4)	P	PM2, PP1, PP5 (VUS)
1.12, trio	TSC	TSC2 c.2221-126C>T, p.?, chr16:2122724C>T	499/1072 (47%)	*De novo*, creates cryptic donor site	LP	PM2, PS2, PP5 (VUS)
1.13, trio	TSC	*TSC2* c.2369_2371del, p.(Tyr790del), chr16:2124214_2124216del	601/1122 (54%)	In-frame deletion, *de novo*; 1 × ClinVar: no assertion provided; functional study (see Supplementary Information, Figure S2)	LP	PM2, PS2, PS3, PP5 (VUS)
1.14, 2 DNAs	TSC	*TSC2* c.2525del, p.(Pro842Hisfs^∗^52), chr16:2124370del	Blood: 2007/4160 (48%) SEGA: 738/1455 (51%)	frameshift; 2 × ClinVar	P	PM2, PP5, PVS1 (VUS)
1.15	TSC	*TSC2* c.2967-460G>A, r.2966ins2967-458_2967-263, p.(Ser989Argfs^∗^82), chr16:2128573G>A	1164/2268 (51%)	Creates cryptic acceptor site; RNA^1.^, cosegregation	novel	PM2 (VUS)
1.16	n/a	*TSC2* c.3690del, p.(Glu1230Aspfs^∗^25), chr16:2131675del	815/1906 (43%)	Frameshift	P	PM2, PVS1, PP5 (LP)
1.17, trio	TSC	*TSC2* c.4006-11G>A, p.?, chr16:2134217G>A	1182/2246 (53%)	Disrupts acceptor site; RNA^1.^, *de novo*	novel	PM2, PS2, PP3 (VUS)
1.18	?	*TSC2* c.4490C>G p.(Pro1497Arg), chr16:2134713C>G	70/150 (47%)	Missense; functional study (LOVD); 1 × ClinVar: no assertion provided	P	PM2, PM5, PS3, PP3, PP5 (LP)
1.19	TSC	*TSC2* c.4544_4547del, p.(Asn1515Serfs^∗^60), chr16:2135002_2135005del	1223/2518 (49%)	Frameshift; 3 × ClinVar	P	PVS1, PM2, PP5 (LP)
1.20, duo	TSC	*TSC2* c.4663-3C>G, p.?, chr16:2136191C>G	1664/3753 (44%)	Shifts acceptor site -2 bp; 1 × ClinVar: no assertion provided	LP	PM2, PP3, PP5 (VUS)
1.21	n/a	*TSC2* c.4842_4844del p.(Ile1614del), chr16:2136373_2136375del	1224/2354 (52%)	In-frame deletion; functional study (see Supplementary Information, Figure S2); 3 × ClinVar: LP	P	PM2, PS3, PP5 (VUS)
1.22	TSC	*TSC2* c.5238_5255del p.(His1746_Arg1751del), chr16:2138295_2138312del	89/202 (44%)	In-frame deletion; functional study	P	PM2, PS3, PP5 (VUS)
1.23	TSC	*TSC1* c.149T>C, p.(Leu50Pro), chr16:135802649A>G	176/342 (51%)	Missense; functional study (LOVD); 1 × ClinVar	P/LP	PM2, PS3, PP5 (VUS)
1.24, trio	TSC	*TSC1* c.363+666T>A, r.363ins68, p.(Met122Aspfs^∗^24), chr16:135800308A>T	5874/13761 (43%)	Creates cryptic acceptor site; *de novo*, RNA^1.^	novel	PM2, PP3, PS2, PS3 (VUS)
1.25	TSC	*TSC1* c.1431_1434del, p.(Glu478Lysfs^∗^53), chr16:135782122_135782125del	123/233 (53%)	Frameshift; 3 × ClinVar	P	PVS1, PM2, PP5 (LP)
1.26, duo	TSC	*TSC1* c.1498C>T, p.(Arg500^∗^), chr16:135781467G>A	1824/4006 (46%)	Stopgain, *de novo*; 4 × ClinVar	P	PVS1,PS2, PM2, PP5 (LP)
1.27, duo	TSC	*TSC1* c.1498C>T, p.(Arg500^∗^), chr9:135781467G>A	1094/2137 (51%)	Stopgain, *de novo*; 4 × ClinVar	P	PVS1, PS2, PM2, PP5 (LP)
1.28, trio	TSC	*TSC1* c.1717C>T, (p.Gln573^∗^), chr9:135781248G>A	2449/4797 (51%)	Stopgain, *de novo*; 1 × ClinVar	P	PVS1, PS2, PM2, PP5 (LP)
1.29	TSC	*TSC1* c.1997+1G>A, p.?, chr9:135780967C>T	1008/2204 (46%)	Destroys donor site; 2 × ClinVar	P	PM2, PP3, PP5 (VUS)

**Table 2:** Inactivating post-zygotic *TSC1* and *TSC2* variants identified using HaloPlex custom capture NGS. Individuals fulfilling the clinical criteria for definite TSC [3] are indicated with “TSC”; those fulfilling only criteria for possible TSC are indicated with “?”; individuals for whom clinical information was not available to us are indicated with “n/a.” VAF, variant allele frequency, refers to the proportion of reads containing the corresponding variant. ^#^For large (> 150 bp) deletions, the VAF was estimated from the allele counts for informative benign variants within the deleted region (data not shown). Cases for which multiple family members or DNA samples were tested are indicated. Evidence for effects on pre-mRNA splicing was obtained by analysis of subject RNA isolated from peripheral blood (RNA^1.^) and/or by *in vitro* exon trap assay (RNA^3^; see Supplementary Information Tables S7 and S8). For functional studies please refer to the *TSC2* Leiden Open Variation Database (LOVD; www.lovd.nl/TSC2). ClinVar (pathogenic, unless stated otherwise), the LOVD, and gnomAD were accessed on 13/2/2023. P: pathogenic; LP: likely pathogenic; VUS: variant of uncertain clinical significance; LB: likely benign. Variants were classified according to the American College of Medical Genetics and Genomics (ACMG) criteria [15] using the ALAMUT Visual Plus (version 1.7) software package.

Subject	Clinical diagnosis	Variant hg19 (GRCh37); NG_005895.1, NM_000548.3(TSC2); NG_012386.1, NM_000368.4(TSC1)	VAF (%)	Evidence for pathogenicity	LOVD	ACMG
2.1	TSC	*TSC2* c.139_140del, p.(Glu47Thrfs^∗^19), chr16:2098753_2098754del	35/295 (12%)	Frameshift; 3 × ClinVar	P	PVS1, PM2, PP5 (LP)
2.2	n/a	*TSC2* c.139-2A>G, p.?, chr16:2100399A>G	128/1714 (7%)	Destroys acceptor site; 1 × ClinVar	P	PM2,PP3, PP5 (VUS)
2.3	TSC	*TSC2* c.268C>T, p.(Gln90^∗^), chr16:2103385C>T	67/1000 (7%)	Stopgain; 4 × ClinVar	P	PVS1, PM2, PP5 (LP)
2.4	?	*TSC2* c.268C>T, p.(Gln90^∗^), chr16:2103385C>T	53/993 (5%)	Stopgain; 4 × ClinVar	P	PVS1, PM2, PP5 (LP)
2.5	n/a	*TSC2* c.299del, p.(Ala100Glyfs^∗^6), chr16:2103416del	21/1197 (2%)	Frameshift	P	PVS1, PM2, PP5 (LP)
2.6	TSC	*TSC2* c.352dup, p.(Val118Glyfs^∗^8), chr16:2104312dup	23/1133 (2%)	Frameshift, 2 × ClinVar	P	PVS1, PM2, PP5 (LP)
2.7, duo	TSC	*TSC2* c.848+281C>T, p.? chr16:2107460C>T	70/1830 (4%)	Creates a cryptic donor site; 3 × ClinVar (affected parent of subject 1.5 (Table 1)	P	PP1, PP3, PM2, PP5 (VUS)
2.8	TSC	*TSC2* c.848+281C>T, p.? chr16:2107460C>T	183/642 (29%)	Creates a cryptic donor site; 3 × ClinVar	P	PP3, PM2, PP5 (VUS)
2.9	TSC	*TSC2* c.976-15G>A, p.?, chr16:2110656G>A	16/412 (4%)	Creates a cryptic acceptor site; RNA^1.^; 3 × ClinVar	P	PM2, PP3, PP5 (VUS)
2.10	n/a	*TSC2* c.990_1005del, p.(Asn331Metfs^∗^27), chr16:2110685_2110700del	248/2317 (11%)	Frameshift	novel	PVS1, PM2 (LP)
2.11	TSC	*TSC2* c.1120-28_1120-10del, p.?, chr16:2111845_2111863del	103/1416 (7%)	Destroys acceptor site	novel	PM2, PP3, PM2 (VUS)
2.12	TSC	*TSC2* c.1210C>T, p.(Gln404^∗^), chr16:2111962C>T	169/1509 (11%)	Stopgain; 2 × ClinVar	P	PVS1,PM2, PP5 (LP)
2.13	TSC	*TSC2* c.1221C>A, p.(Tyr407^∗^), chr16:2111973C>A	66/1929 (3%)	Nonsense; 1 × ClinVar	P	PVS1, PM2, PP5 (LP)
2.14, trio	TSC	*TSC2* c.1258-1G>A, p.?, chr16:2112497G>A	596/7538 (8%)	Disrupts acceptor site; 1 × ClinVar: no assertion provided	P	PP3, PM2, PP5 (VUS)
2.15, trio	TSC	*TSC2* c.1361+1G>A, p.?, chr16:2112602G>A	46/1439 (3%)	Destroys donor site; 2 × ClinVar	P	PP3, PM2. PP5 (VUS)
2.16	TSC	*TSC2* c.1372C>T p.(Arg458^∗^), chr16:2112983C>T	21/505 (4%)	Nonsense: 4 × ClinVar	P	PVS1, PM2, PP5 (LP)
2.17	n/a	*TSC2* c.1492G>T p.(Glu498^∗^), chr16:2114321G>T	226/990 (23%)	Nonsense; 1 × ClinVar	novel	PVS1, PM2, PP5 (LP)
2.18	TSC	*TSC2* c.1636del, p.(Glu546Lysfs^∗^15), chr16:2115556del	22/910 (2%)	Frameshift	P/LP	PVS1, PS3, PM2, PP3, PP5 (LP)
2.19	TSC	*TSC2* c.1831C>T, p.(Arg611Trp), chr16:2120571C>T	24/542 (4%)	Missense; functional study (LOVD); 5 × ClinVar	P/LP	PS3, PM2, PP3, PP5 (LP)
2.20	TSC	TSC2 c.1831C>T, p.(Arg611Trp), chr16:2120571C>T	60/2193 (3%)	Missense; functional study (LOVD); 5 × ClinVar	P/LP	PS3, PM2, PP3, PP5 (LP)
2.21	TSC	*TSC2* c.1832G>A, p.(Arg611Gln), chr16:2120572G>A	19/1305 (1%)	Missense; functional study (LOVD); 6 × ClinVar	P	PS3, PM2, PP3, PP5 (LP)
2.22, trio	TSC	*TSC2* c.1852del p.(Leu618Cysfs^∗^80), chr16:2121523del	102/545 (19%)	Frameshift	P	PVS1, PM2, PP5 (LP)
2.23, 2 DNAs	TSC	*TSC2* c.2108G>A, p.(Trp703^∗^), chr16:2122252G>A	blood 1: 119/1459 (8%) blood 2: 49/690 (7%)	Stopgain; 2 × ClinVar	P	PVS1, PM2, PP5 (LP)
2.24	TSC	*TSC2* c.2119_2120ins[2098_2119;GTCT], p.(Lys707Argfs^∗^3), chr16:2122263insGAGTCTGACTGGAAGGTGCTGAGTCT	19/1056 (2%)	Frameshift	novel	PVS1, PM2 (LP)
2.25	TSC	*TSC2* c.2251C>T p.(Arg751^∗^), chr16:2122880C>T	54/1761 (3%)	Stopgain; 4 × ClinVar	P	PVS1, PM2, PP5 (LP)
2.26, trio	TSC	*TSC2* c.2590C>T, p.(Gln864^∗^), chr16:2125844C>T	83/2359 (4%)	Stopgain; 2 × ClinVar; confirmed in DNA from an angiofibroma by DD PCR	P	PVS1, PM2, PP5 (LP)
2.27	?	*TSC2* c.2590_2593dup, p.(Tyr865fs^∗^19), chr16:2125844_2125847dup	10/1698 (0.6%)	Frameshift; variant confirmed in DNA isolated from a facial angiofibroma: 41/888 (4%) (R. Oegema, personal communication)	novel	PVS1, PM2 (LP)
2.28	TSC	*TSC2* c.2687G>A, p.(Trp896^∗^), chr16:2126116G>A	30/1796 (2%)	Stopgain; 1 × ClinVar	P	PVS1, PM2, PP5 (LP)
2.29	?	*TSC2* c.2713C>T, p.(Arg905Trp), chr16:2126142C>T	8/706 (1%)	Missense; functional study (LOVD); 4 × ClinVar	P	PS3, PM2, PP3, PP5 (LP)
2.30, trio	TSC	*TSC2* c.2742G>A, p.(Lys914=), chr16:2126171G>A	206/1536 (13%)	Destroys donor site; 2 × ClinVar: LP	P/LP/VUS	PM2, PP5, PP3, BP4 (VUS)
2.31	TSC	*TSC2* c.2838-122G>A, r.2837ins120fs, p.( Ser949Argins4^∗^), chr16:2127477G>A	133/368 (36%)	Creates cryptic acceptor site; RNA [11], RNA^3.^; 3 × ClinVar	LP	PM2, PS3, PP3, PP5 (VUS)
2.32	TSC	TSC2 c.2838-122G>A, r.2837ins120fs, p.( Ser949Argins4^∗^), chr16:2127477G>A	172/551 (31%)	Creates cryptic acceptor site; RNA [11], RNA^3.^; 3 × ClinVar	LP	PM2, PS3, PP3, PP5 (VUS)
2.33, duo	TSC	*TSC2* c.2838-122G>A, r.2837ins120fs, p.(Ser949Argins4^∗^), chr16:2127477G>A	1426/10593 (15%)	Creates cryptic acceptor site; RNA [11], RNA^3.^; 3 × ClinVar	LP	PM2, PS3, PP3, PP5 (VUS)
2.34, trio	TSC	*TSC2* c.2838-122G>A, r.2837ins120fs, p.( Ser949Argins4^∗^), chr16:2127477G>A	1130/4097 (15%)	Creates cryptic acceptor site; RNA [11], RNA^3.^; 3 × ClinVar	LP	PM2, PS3, PP3, PP5 (VUS)
2.35	?	*TSC2* c.2838-122G>A, r.2837ins120fs, p.( Ser949Argins4^∗^), chr16:2127477G>A	123/625 (20%)	Creates cryptic acceptor site; RNA [11], RNA^3.^; 3 × ClinVar	LP	PM2, PS3, PP3, PP5 (VUS)
2.36	TSC	*TSC2* c.3094C>T, p.(Arg1032^∗^), chr16:2129160C>T	12/636 (2%)	Stopgain; 3 × ClinVar	P	PVS1, PM2, PP5 (LP)
2.37	TSC	TSC2 c.3412C>T p.(Arg1138^∗^), chr16:2130180C>T	15/1444 (1%)	Stopgain; 4 × ClinVar	P	PVS1, PM2, PP5 (LP)
2.38	TSC	*TSC2* c.3412C>T p.(Arg1138^∗^), chr16:2130180C>T	33/201 (16%)	Stopgain; 4 × ClinVar	P	PVS1, PM2, PP5 (LP)
2.39, trio	TSC	*TSC2* c.3520del, p.(Arg1174Glyfs^∗^17), chr16:2130288del	105/3175 (3%)	Frameshift; 1 × ClinVar	P	PVS1, PM2, PP5 (LP)
2.40	TSC	*TSC2* c.3696dup, p.(Asn1233^∗^), chr16:2131681dup	72/2640 (3%)	Stopgain; 3 × ClinVar	P	PVS1, PM2, PP5 (LP)
2.41, trio	TSC	*TSC2* c.4351dup p.(Arg1451Profs^∗^73), chr16:2134574dup	64/880 (7%)	Frameshift; 2 × ClinVar	P	PVS1, PM2, PP5 (LP)
2.42	?	*TSC2* c.4488_4491C[6], p.(Ser1498Profs^∗^79), chr16:2134713_2134714dup	132/1217 (11%)	Frameshift	novel	PVS1, PM2 (LP)
2.43	TSC	*TSC2* c.4490C>T, p.(Pro1497Leu), chr16:2134713C>T	7/55 (13%)	Missense; functional study (see Supplementary Information Figure S2)	P	PVS1, PM2, PP5 (LP)
2.44	TSC	*TSC2* c.4537G>T, p.(Glu1513^∗^), chr16:2134995G>T	69/521 (13%)	Stopgain	P	PVS1, PM2, PP5 (LP)
2.45, trio	TSC	*TSC2* c.4959C>A, p.(Ser1653=), chr16:2136842C>A	270/1100 (25%)	Creates cryptic donor site; RNA^3.^; 1 × ClinVar: VUS	LB	PM2, BP4, PS3, PVS1 (LP)
2.46, 2 DNAs	TSC	*TSC2* c.5024C>T p.(Pro1675Leu), chr16:2137898C>T	blood: 106/592 (18%) cultured normal skin fibroblasts: 133/556 (24%)	Missense; functional study (LOVD); 4 × ClinVar	P	PM2, PS3, PP5 (LP)
2.47, trio	TSC	*TSC2* c.5069-2A>G, p.?, chr16:2138047A>G	17/551 (3%)	Disrupts acceptor site; 2 × ClinVar	P	PM2, PP3, PP5 (VUS)
2.48, trio	TSC	*TSC2* c.5183_5184insGCCG, p.(Ser1728Argfs^∗^48), chr16:2138250_2138251insGCCG	295/1487 (20%)	Frameshift	novel	PVS1, PM2 (LP)
2.49, trio	TSC	*TSC2* c.5227C>T, p.(Arg1743Trp), chr16:2138294C>T	247/2031 (12%)	Missense; functional study (LOVD); 5 × ClinVar	P	PM1, PM2, PM5, PS3, PP3, PP5 (LP)
2.50, trio	TSC	*TSC2* c.5228G>A, p.(Arg1743Gln), chr16:2138295G>A	271/4114 (7%)	Missense; functional study (LOVD); 3 × ClinVar: LP	P	PM1, PM2, PM5, PS3, PP3, PP5 (LP)
2.51	TSC	*TSC2* c.5228G>A, p.(Arg1743Gln), chr16:2138295G>A	12/71 (17%)	Missense; functional study (LOVD); 3 × ClinVar: LP	P	PM1, PM2, PM5, PS3, PP3, PP5 (LP)
2.52, duo	TSC	*TSC2* c.(?_-106)_(?_1362-50)del, p.?, chr16: (?_2097990)_(?_2112923)del	(~15%)^#^	Large deletion (~24 kb); confirmed by SNP array, MLPA	novel	PVS1, PM2 (LP)
2.53	TSC	*TSC2* c.(?_-106)_(^∗^102_?)del, p.?, chr16: (?_2097990)_(2138713_?)del	(~10%)^#^	Large deletion (~52 kb); confirmed by MLPA	P	PVS1, PM2, PP5 (LP)
2.54	TSC	*TSC1* c.587C>T p.(Pro196Leu), chr9:135797282G>A	137/803 (17%)	Missense; functional study (see Supplementary Information Figure S2)	LP	PS3, PM2, PP5 (VUS)

**Table 3:** *TSC1* and *TSC2* variants of uncertain clinical significance (VUS), lesion-specific variants, and unconfirmed findings identified using HaloPlex custom capture NGS. Individuals fulfilling the clinical criteria for definite TSC [3] are indicated with “TSC”; those fulfilling only criteria for possible TSC are indicated with “?.” VAF: variant allele frequency, refers to the proportion of reads containing the corresponding variant. Cases for which multiple DNA samples or family members were tested are indicated. Evidence for effects on pre-mRNA splicing was obtained by analysis of subject RNA isolated from peripheral blood (RNA^1.^) and/or by in vitro exon trap assay (RNA^3^; see Supplementary Information Tables S7 and S8). ClinVar, Leiden Open Variation Database (LOVD), and gnomAD were accessed on 13/2/2023. P: pathogenic; LP: likely pathogenic; VUS: variant of uncertain clinical significance; LB: likely benign. Variants were classified according to the American College of Medical Genetics and Genomics (ACMG) criteria [15] using the ALAMUT Visual Plus (version 1.7) software package. Individuals 3.23-3.72 remained NMI after HaloPlex analysis (see Supplementary Information, Table S6).

Subject	Diagnosis	Variant hg19 (GRCh37); NG_005895.1, NM_000548.3(TSC2); NG_012386.1, NM_000368.4(TSC1)	VAF (%)	Evidence for/against pathogenicity	LOVD	ACMG
3.1	TSC	*TSC2* c.226-1222G>T, p.?, chr16:2102121G>T	590/1359 (43%)	2/140086 gnomAD; creates cryptic donor	Novel	PM2, PP3 (VUS)
3.2	?	*TSC2* c.337-183G>A, p.?, chr16:2104114G>A	286/704 (41%)	Novel	Novel	PM2, BP4 (VUS)
		*TSC2* c.3397+230C>T, p.?, chr16:2129900C>T	334/691 (48%)	1/31352 gnomAD	Novel	PM2, BP4 (VUS)
3.3	?	*TSC2* c.482-400T>C, p.?, chr16:2105003T>C	516/938 (55%)	Novel	Novel	PM2, BP4 (VUS)
		*TSC2* c.1716+284C>G, p.?, chr16:2115920C>G	52/876 (6%)	Novel	Novel	PM2, BP4 (VUS)
3.4, trio	TSC	*TSC2* c.529dup p.(Leu177Profs^∗^12) chr16:2105450dup	2/84 (2%)	Frameshift; variant unconfirmed	Novel	PVS1, PM2 (LP)
3.5, 2 DNAs	TSC	*TSC2* c.599+4A>G, p.?, chr16:2105524A>G	blood: 0/759 (0%) SEGA (FFPE): 12/39 (30%)	Destroys donor site: RNA^1., 3.^; variant unconfirmed	LP/VUS	PM2, PP3, PS3 (VUS)
3.6, trio	TSC	*TSC2* c.1600-3_1656del, p.?, chr16:2115517_2115576del	2/652 (0.3%)	Destroys acceptor site	Novel	PP3, PM2 (VUS)
3.7, 2 DNAs	?	*TSC2* c.4375C>T p.(Arg1459^∗^), chr16:2134598C>T	blood: 1/1069 (0%) SEGA: 299/568 (53%)	Stopgain; 3 x ClinVar (gnomAD 1/31396)	*P*	PVS1, PM2, PP5 (LP)
3.9	?	*TSC2* c.1717-785_1717-784del, p.?, chr16:2119672_2119673del	64/131 (49%)	Movel; no effect on splicing: RNA^1.^	Novel	PM2, BS3, BP4 (VUS)
3.10	?	*TSC2* c.2859del, p.(Lys954Asnfs^∗^4), chr16:2127620del	8/902 (1%)	Novel; frameshift in alternatively spliced exon	P/LB	PM2 (VUS)
3.11	?	TSC2 c.4006-118G>A, p.?, chr16:2134111G>A	178/622 (29%)	Novel; no effect on splicing: RNA^3.^	Novel	PM2, BP4, BS3 (VUS)
3.12	TSC	*TSC2* c.5161-32A>C, p.?, chr16:2138196A>C	286/616 (46%)	Novel; no effect on splicing: RNA^3.^	Novel	PM2 (VUS)
3.13	?	*TSC1* c.363+334C>T, p.?, chr9:135800640G>A	366/881 (42%)	Novel; no effect on splicing: RNA^3.^	Novel	PM2, BP4, BS3 (VUS)
3.14	TSC	*TSC1* c.363+633_363+634delinsTT, p.?, chr9:135800340_135800341delinsAA	165/373 (44%)	Novel; predicted to create a cryptic acceptor	Novel	PM2, PP3 (VUS)
3.15	TSC	*TSC1* c.1264-728T>G, p.?, chr9:135783485A>C	182/354 (51%)	Novel; predicted to create cryptic acceptor site; no effect on splicing: RNA^3.^	Novel	PM2, PS3, BS3 (VUS)
3.16	?	*TSC1* c.1997+17C>G, p.?, chr9:135780951G>C	534/1428 (37%)	No effect on splicing: RNA^3.^; 1 x ClinVar: likely benign	LB/VUS	PM2, BP6, BS3 (VUS)
3.17	?	*TSC1* c.2392-110G>C, p.?, chr9:135777196C>G	1321/3151 (42%)	Novel; no effect on splicing: RNA^3.^	Novel	PM2, BS3 (VUS)
3.19, 3 DNAs	?	*TSC2* c.5024C>T p.(Pro1675Leu), chr16:2137898C>T	blood: 1/2200 (0%); cultured normal skin fibroblasts: 0/2156 (0%); Shagreen patch: 46/2242 (2%)	Missense; functional study (LOVD); 4 x ClinVar; 15 Mb deletion identified in DNA isolated from Shagreen patch, VAF ~15% (E. Legius, personal communication)	P	PM1, PM2, PM5, PP3, PS3, PP5 (LP)
3.20, 2 DNAs	TSC	*TSC2* c.^∗^141G>T, p.?; NM_001009944.2(PKD1):c.^∗^976C>A, p.? chr16:2138752G>T	blood: 69/348 (20%); angiofibroma: 276/1216 (23%)	Novel	Novel	PM2 (VUS)
		*TSC2* c.1331del, p.(Asn444Thrfs^∗^5), chr16:2112570del	0/466 (0%) 22/642 (3%)	Frameshift	*P*	PVS1, PM2, PP5 (LP)
3.21, trio	TSC	*TSC2* c.5200G>T p.(Asp1734Tyr), chr16:2138267G>T	8/311 (3%)	Novel, missense; unconfirmed	Novel	PM2 (VUS)
3.22	TSC	*TSC1* c.1439-57G>T, p.?, chr9:135781583C>A	6/543 (1%)	Novel, predicted to create a cryptic acceptor site; variant unconfirmed	Novel	PM2, PP3 (VUS)

## Data Availability

Variants have been deposited in the *TSC1* and *TSC2* LOVD [https://databases.lovd.nl/shared/genes/TSC1 and https://databases.lovd.nl/shared/genes/TSC2]. Primer sequences are available on request. The data that support the findings of this study are available from the corresponding authors, with the exception of primary patient sequencing data, as they are derived from patient samples with unique variants that are impossible to guarantee anonymity for. Our institutional guidelines do not allow sharing these raw data, as this is not part of the patient consent procedure.

## Supplementary Materials

Supplementary Materials The Supplementary Information consists of extended methods and the following 5 figures and 9 tables: Figure S1: comparison of the variant allele frequencies (VAF) for selected TSC NMI subjects. Figure S2: Functional assessment of TSC1 and TSC2 variants identified using HaloPlex custom capture NGS. Figure S3. Detection of large (>150 bp) deletions using *z*-scores. Figure S4: Segregation of the NM_000548.3(TSC2):c.1947-23A>G variant. Figure S5: Comparison of read depths per subject. Supplementary Information Table S1: HaloPlex and HaloPlex HS custom capture design characteristics. Supplementary Information Table S2: HaloPlex and HaloPlex HS custom capture data yield and alignment statistics. Supplementary Information Table S3: HaloPlex and HaloPlex HS NGS custom capture coverage per subject. Supplementary Table S4: Clinical features of TSC NMI subjects for whom inactivating, likely germline *TSC1* and *TSC2* variants were identified using HaloPlex custom capture NGS. Supplementary Table S5: Clinical features of TSC NMI subjects for whom inactivating, post-zygotic *TSC1* and *TSC2* variants were identified using HaloPlex custom capture NGS. Supplementary Table S6: Clinical features of TSC NMI subjects with *TSC1* and *TSC2* variants of uncertain clinical significance (VUS), lesion-specific variants, and/or unconfirmed findings. Supplementary Information Table S7: Exon trap analysis of *TSC1* variants of uncertain clinical significance. Supplementary Information Table S8: Exon trap analysis of *TSC2* variants of uncertain clinical significance. Supplementary Information Table S9: Single nucleotide variant (SNV) allele frequencies for copy number variant detection.
