# Supplementary material for: Targeted Genomic Sequencing of TSC1 and TSC2 Reveals Causal Variants in Individuals for Whom Previous Genetic Testing for Tuberous Sclerosis Complex Was Normal
Source: Hum Mutat. 2023 Jul 13;2023:4899372. doi: 10.1155/2023/4899372 (PMC11918493; doi:10.1155/2023/4899372)
Supplement: Supplementary Materials — The Supplementary Information consists of extended methods and the following 5 figures and 9 tables: Figure S1: comparison of the variant allele frequencies (VAF) for selected TSC NMI subjects. Figure S2: Functional assessment of TSC1 and TSC2 variants identified using HaloPlex custom capture NGS. Figure S3. Detection of large (>150 bp) deletions using z-scores. Figure S4: Segregation of the NM_000548.3(TSC2):c.1947-23A>G variant. Figure S5: Comparison of read depths per subject. Supplementary Information Table S1: HaloPlex and HaloPlex HS custom capture design characteristics. Supplementary Information Table S2: HaloPlex and HaloPlex HS custom capture data yield and alignment statistics. Supplementary Information Table S3: HaloPlex and HaloPlex HS NGS custom capture coverage per subject. Supplementary Table S4: Clinical features of TSC NMI subjects for whom inactivating, likely germline TSC1 and TSC2 variants were identified using HaloPlex custom capture NGS. Supplementary Table S5: Clinical features of TSC NMI subjects for whom inactivating, post-zygotic TSC1 and TSC2 variants were identified using HaloPlex custom capture NGS. Supplementary Table S6: Clinical features of TSC NMI subjects with TSC1 and TSC2 variants of uncertain clinical significance (VUS), lesion-specific variants, and/or unconfirmed findings. Supplementary Information Table S7: Exon trap analysis of TSC1 variants of uncertain clinical significance. Supplementary Information Table S8: Exon trap analysis of TSC2 variants of uncertain clinical significance. Supplementary Information Table S9: Single nucleotide variant (SNV) allele frequencies for copy number variant detection. [file 4899372.f1.zip › SuppInfo_30523.pdf]

Supplementary Information

Targeted genomic sequencing of *TSC1* and *TSC2* reveals causal variants in individuals for whom previous genetic testing for tuberous sclerosis complex was normal.

Hannah D. West, Mark Nellist, Rutger W.W. Brouwer, Mirjam C.G.N. van den Hout-van Vroonhoven, Luiz Gustavo Dufner de Almeida, Femke Hendriks, Peter Elfferich, Meera Raja, Peter Giles, Rosa M. Alfano, Angela Peron, Yves Sznajer, Liesbeth De Waele, Anna Jansen, Marije Koopmans, Anneke Kievit, Laura S. Farach, Hope Northrup, Julian R. Sampson, Laura E. Thomas and Wilfred F.J. van IJcken

Supplementary Methods

Patient Cohort

Subjects were referred for testing to the Erasmus Medical Center (EMC), Rotterdam, the Netherlands or the Institute of Medical Genetics, Cardiff, United Kingdom because of a diagnosis of definite or possible TSC [1], or who were suspected of TSC but had inadequate clinical details for classification, and were TSC 'no mutation identified' (NMI) after diagnostic testing of *TSC1* and *TSC2* that included sequencing of all coding exons and intron-exon boundaries by PCR and Sanger sequencing approaches, and multiplex ligation probe amplification (MLPA) for detection of large rearrangements. To examine the ability of our approach to detect large rearrangements, we included 4 control samples containing large rearrangements affecting the *TSC2* locus (see below). The study was approved by the NHS research ethics committee for Wales (REC 11WA0276). All individuals tested in Rotterdam had requested genetic testing of *TSC1* and *TSC2* for diagnostic purposes and informed consent was provided by all subjects, as required by the institutional review board of the EMC (METC-2012-387) and the referring institution, and according to standard diagnostic protocols.

DNA and RNA isolation

Genomic DNA and total RNA were extracted from peripheral blood, affected and normal skin samples and/or cultured skin fibroblasts using standard procedures. DNA quality and concentration were checked with the Quant-iT PicoGreen dsDNA Kit (Invitrogen, Carlsbad, USA).

HaloPlex custom capture NGS

All subjects were analysed using customised HaloPlex or HaloPlex HS target enrichment assays (Agilent Technologies, Santa Clara, USA) encompassing the *TSC1* and *TSC2* genomic loci [2]. Care was taken to ensure that the *PKD1* repeat region downstream of *TSC2* was excluded.

We used 5 different HaloPlex designs, A - E. The HaloPlex approach uses different combinations of restriction enzymes to fragment the target genomic DNA and probes that hybridize to both ends of these fragments. This results in multiple, overlapping amplicons across most of the target region. Design characteristics, data yield and alignment statistics per design are shown in Tables S1 and S2. To try and attain complete coverage of both loci a series of low stringency probes were included in designs A and B. This was not successful. Coverage was still incomplete and the quality of the sequence data across the regions corresponding to the low stringency probes was often of low quality, resulting in false variant calls. For design E the repeat and low complexity regions were excluded from the HaloPlex array to reduce the number of false calls and improve coverage elsewhere. To increase the sensitivity of detection for exonic regions we increased the proportion of exonic probes for designs A, B and E.

Assays were performed according to the manufacturer’s instructions using 200 ng and 50 ng genomic DNA per subject for the standard HaloPlex and HaloPlex HS assay respectively. Libraries were pooled and run on an Illumina HiSeq 2500 using the paired-end 100 base pair (bp) sequencing protocol, an Illumina MiSeq using the paired-end 150 bp V2 protocol, or an Illumina MiSeq using the paired-end 300 bp V3 protocol. For the HaloPlex HS samples, unique molecular identifiers (UMI) enabled the removal of PCR duplicates.

Bioinformatic analysis

Adapter sequences were trimmed prior to alignment and reads were mapped to reference sequences NG_012386.1 (TSC1) and NG_005895.1 (TSC2) of build GRCh37 (hg19) of the human genome using the NIMBUS pipeline, as described previously [2, 3]. Single nucleotide substitution and insertion/deletion (indel) variants were called using in-house software. For HaloPlex HS, 4 sequence reads per DNA molecule were generated: 2 of 100 or 300 bp, 1 index read and 1 UMI read. The index was used to determine to which sample the reads belonged, the UMI was used to determine the specific DNA molecule. Reads in the same genomic region with the same UMI originated from the same DNA molecule prior to amplification. The data reads were derived from the actual *TSC1* and *TSC2* genomic loci. Data was demultiplexed using bcl2fastq2 (Illumina) and converted to FastQ files. During processing, the UMI read (I2) was encoded in the FastQ headers of the data reads (R1 and R2). Reads were trimmed up to the expected HaloPlex adapter sequences, CTGTCTCTTATA and GAGATCGGAAGAG, followed by removal of 5' Ns from read 1 and 5' Ns and Gs from read 2. The trimmed reads were aligned to the human genome (hg19) using BWA mem whereafter the UMIs extracted from the read names were added to the the um tag in the resulting SAM file. Data were processed through Samtools addreplacerg and Samtools sort, resulting in a BAM file with coordinate sorted alignments. The alignments were sorted on read name and mate pairs were fixed by samtools sort -n and samtools fixmate respectively. The mate pair fixed files were sorted on coordinate again and merged per sample resulting in one BAM file per sample with fixed mate pairs ready for follow-up analysis. Consensus alignments were determined from the BAM files using pyngs umi-consensus (https://github.com/erasmus-center-for-biomics/pyngs/) for alignments with a distance of < 20 bp and -consensus was added to the sample name in the BAM files using samtools reheader. Adding the -consensus suffix allowed the consensus alignments to be distinguished from the original alignments during variant calling. The median read depth and range per subject sample across the target regions are shown in Table S3.

Variants were called per sample by processing the original and consensus alignments with bcftools mpileup, pyngs_tools vcf-allele-filter and bcftools call. Multiallelic variants were split using bcftools norm whereafter all variants were merged using bcftools merge and chromosomes renamed to the GRCh37 standard. Variants were annotated with gnomAD variant frequencies before remapping the chromosome names to the hg19 standard. Annotations were added as separate fields by py_ngs vcf-parse-annotations. Variants were called together in all samples on the positions of the annotated VCF file by bcftools mpilelup using a minimum mapping quality of 10 and a maximum depth of 10000. Variant frequencies were added with py_ngs vcf-allele-filter and (diploid) genotypes were determined with bcftools call. The resulting variants were annotated with the previously generated annotations using bcftools annotate and converted to a tabular file using bcftools norm followed by bcftools query. Variants were included in the combined variant list if a variant occurred in a single sample with an allele frequency of at least 1% and at least 5 alignments with the alternate allele. This approach was chosen to filter out the sequence errors while keeping potential variants that occurred in other samples.

Variants were visualised and manually interrogated with the Integrative Genomics Viewer (IGV, http://www.broadinstitute.org/igv/), defined according to reference sequences NG_012386.1 (TSC1) and NG_005895.1 (TSC2), and reference transcripts NM_000548.3 (TSC2) and NM_000368.4 (TSC1), and compared with variants listed in dbSNP, 1000genomes, gnomAD, ExAc and the *TSC1* and *TSC2* LOVD databases*.* To identify variants with potential effects on splicing, the Annovar, Max-EntScan, NNSPLICE, GeneSplicer and Human Splice Finder algorithms in the ALAMUT Visual Plus version 1.7 software package (Sophia Genetics, Bidart, France) were applied.

To identify deletions > 150 bp and other rearrangements that might have prevented fragment capture, we compared the variant allele frequency (VAF) for single nucleotide variants (SNVs) across both loci. We compared the read depths for the different HaloPlex capture fragments per subject DNA using z-scores [4], utilizing a control cohort consisting of the unaffected family members and individuals for whom a pathogenic variant affecting a single or a few nucleotides had already been

identified. Per consensus sample, the read depth at each location was divided by the cumulative read depth yielding a relative read depth. Thereafter, the mean relative read-depth per location was subtracted from the relative read depth in that sample and divided by the standard deviation in relative read depth, resulting in a z-score. Because the z-score method identifies potential differences in copy-number, but not in exact differences, validation by different methods is required. We used MLPA, SNP array analysis and comparison of the VAFs for SNVs across the *TSC1* and *TSC2* loci for validation of the identified changes and to estimate the corresponding VAF (see below, *Validation of identified variants*).

We considered regions with a z-score < -2.5 as indicating a possible deletion and > 2.5 as suggestive of a duplication. As controls we included genomic DNA samples from 4 individuals with known rearrangements at the *TSC2* locus: *TSC2* c.1600-182_2546-349del (10 kb germline deletion); *TSC2* c.976-126_1060del (0.2 kb germline and post-zygotic deletion); and chr16: g.2119485_2513252delins(2119568_2513173inv) (0.4Mb germ-line inversion between *TSC2* and the tubulin epsilon and delta complex 2 gene (*TEDC2*) locus). Both individuals with the *TSC2* c.976-126_1060del deletion were identified using our standard analysis pipeline while the *TSC2* c.1600-182_2546-349del deletion was only identified after the z-score-based comparison of the relative depth of coverage for all amplicons across the *TSC2* locus (Figure S3). We did not find evidence for the 0.4 Mb inversion in the HaloPlex data (not shown), most likely due to the inability to capture the corresponding breakpoint junction fragments and indicating that the HaloPlex methodology is not suitable for the detection of large (> 150 bp) copy neutral rearrangements.

Validation of identified variants

Variants that were identified at high frequency (VAF > 40%) and were therefore likely to be germline changes, were validated using a combination of PCR and Sanger sequencing. Primer sequences and PCR conditions can be provided upon request. Sanger sequencing was performed on an ABI3730 (Applied Biosystems) and analysed using Sequencher software V5.4 (Genecodes) or SeqPilot

(Applied Biosystems). For variants identified at low frequencies (VAF < 40%), allele-specific (AS) PCR, droplet digital (DD) PCR, or Nextera XT-based NGS of specific PCR products was used for validation. AS-PCR was performed according to a standard optimisation protocol, as described previously [2]. Final PCR conditions and primer sequences are available on request. Subject genomic DNA samples were compared to unrelated, non-TSC control samples as well as DNA negative samples. In cases where positive control samples were available, these were also used for assay validation. For DD-PCR, 50 ng genomic DNA was used following the manufacturer’s instructions (Bio-Rad). For subject 2.26, a positive control was generated by site directed mutagenesis (Quikchange, Agilent) of a *TSC2* plasmid. Custom probes and primers were obtained from Bio-Rad (details available upon request). For NGS-based validation, paired-end libraries were prepared using the Nextera XT DNA Sample Preparation kit (Illumina), according to the manufacturer's protocol and sequenced on an Illumina Miseq/Miniseq platform.

For validation of variants predicted to affect pre-mRNA splicing, total RNA was isolated from either subject blood or cultured skin fibroblasts, converted to cDNA using a cDNA synthesis kit (PCR Biosystems), followed by PCR, agarose gel electrophoresis and Sanger sequencing.The effects of missense and in-frame deletion variants on TSC complex function were assessed using standard *in vitro* functional assays, as described previously [5]. In cases where no RNA was available, the potential effects of variants on pre-mRNA splicing were investigated using an *in vitro* exon trap assay, as described previously [5] (Tables S7 and S8).

Large deletions, affecting multiple exons, were validated either by MLPA (MRC Holland, Amsterdam, The Netherlands) or using the GSA-MD-24 global screening single nucleotide polymorphism (SNP) array (Illumina). In addition, the allele frequencies of SNPs across the *TSC1* and *TSC2* genomic loci were compared (Table S9).

References

1. Northrup H, Aronow ME, Bebin EM, Bissler J, Darling TN, de Vries PJ, Frost MD, Fuchs Z, Gosnell ES, Gupta N, Jansen AC, Jóźwiak S, Kingswood JC, Knilans TK, McCormack FX, Pounders A, Roberds SL, Rodriguez-Buritica DF, Roth J, Sampson JR, Sparagana S, Thiele EA, Weiner HL, Wheless JW, Towbin AJ, Krueger DA, International Tuberous Sclerosis Complex Consensus Group (2021) Updated international tuberous sclerosis complex diagnostic criteria and surveillance and management recommendations. Pediatr Neurol 123:50-66.

2. Nellist M, Brouwer RW, Kockx CE, van Veghel-Plandsoen M, Withagen-Hermans C, Prins-Bakker L, Hoogeveen-Westerveld M, Mrsic A, van den Berg MM, Koopmans AE, de Wit MC, Jansen FE, Maat-Kievit AJ, van den Ouweland A, Halley D, de Klein A, van IJcken WF (2015) Targeted Next Generation Sequencing reveals previously unidentified *TSC1* and *TSC2* mutations. BMC Med. Genet. 16:10.

3. Brouwer RWW, van den Hout MCGN, Kockx CEM, Brosens E, Eussen B, de Klein A, Sleutels F, van IJcken WFJ (2018) Nimbus: a design-driven analyses suite for amplicon-based NGS data. Bioinformatics 34:2732-2739.

4. Sinha R, Samaddar S, De RK (2015) CNV-CH: A convex hull based segmentation approach to detect copy number variations (CNV) using Next-Generation Sequencing data. PLoS One 10:e0135895.

5. Dufner Almeida LG, Nanhoe S, Zonta A, Hosseinzadeh M, Kom-Gortat R, Elfferich P, Schaaf G, Kenter A, Kümmel D, Migone N, Povey S, Ekong R, Nellist M (2019) Comparison of the functional and structural characteristics of rare TSC2 variants with clinical and genetic findings. Hum. Mutat. 41:759-773.

Figures

Figure S1: Comparison of the variant allele frequencies (VAF) for selected TSC NMI subjects. For subjects 1.1, 1.10, 1.11, 1.2, 2.31, 2.32, 3.11 and 3.16 a candidate disease-causing variant was identified with a VAF between 30 and 40% (red dots). The VAF for additional variants identified across the corresponding locus are indicated for each subject (black dots). The box indicates subjects 1.10 and 1.11 who are obligate heterozygotes for the *TSC2* c.1947-23A>G variant that segregates with TSC in a 4-generation family (see Figure S4). For these samples the low apparent VAF might be due to inefficient capture of fragments containing the G-allele caused by the removal of a PvuII restriction site used for the generation of the HaloPlex capture fragments.

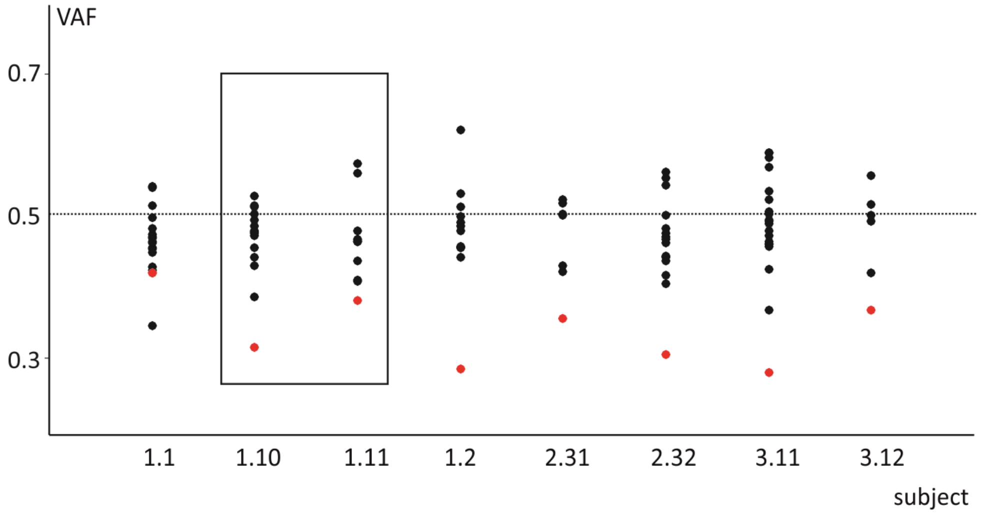

Figure S2: Functional assessment of *TSC1* and *TSC2* variants identified using HaloPlex custom capture NGS.

(A - D) Functional assessment of *TSC1* variants. NM_000368.4(TSC1) c.587C>T p.(Pro196Leu) (subject 2.54, P196L) was expressed in *TSC1* KO HEK 293T cells in 4 independent transfection experiments. Expression of the mean TSC2 (A), TSC1 (B) and S6K (D) signals, and mean T389/S6K ratio (C) are shown. The TSC-associated TSC1 p.Leu117Pro variant (L117P) is shown for comparison.

(E - H) Functional assessment of *TSC2* variants. NM_000548.3(TSC2) c.2369_2371del, p.(Tyr790del) (subject 1.13; 790del); c.4842_4844del, p.(Ile1614del) (subject 1.21, 1614del) and c.4490C>T, p.(Pro1497Leu) (subject 2.43, P1497L) were expressed in *TSC1:TSC2* DKO HEK 293T cells in 4 independent transfection experiments. Expression of the mean TSC2 (E), TSC1 (F) and S6K (H) signals, and mean T389/S6K ratio (G) are shown. The TSC-associated TSC2 p.Arg611Gln variant (R611Q) is shown for comparison.

Dotted lines indicate the signal/ratio for the wild-type control (TSC1 or TSC2; = 1.0); error bars represent the standard error of the mean; an asterisk indicates a significant difference (*P* <0.025, paired Student's t-test) compared to the wild-type control. The T389/S6K ratio was used to estimate TORC1 activity: an increase in the T389/S6K ratio corresponds to increased mechanistic target of rapamycin complex 1 (TORC1) activity and therefore to a loss of TSC complex function. S6K: p70 S6 kinase; T389: threonine 389

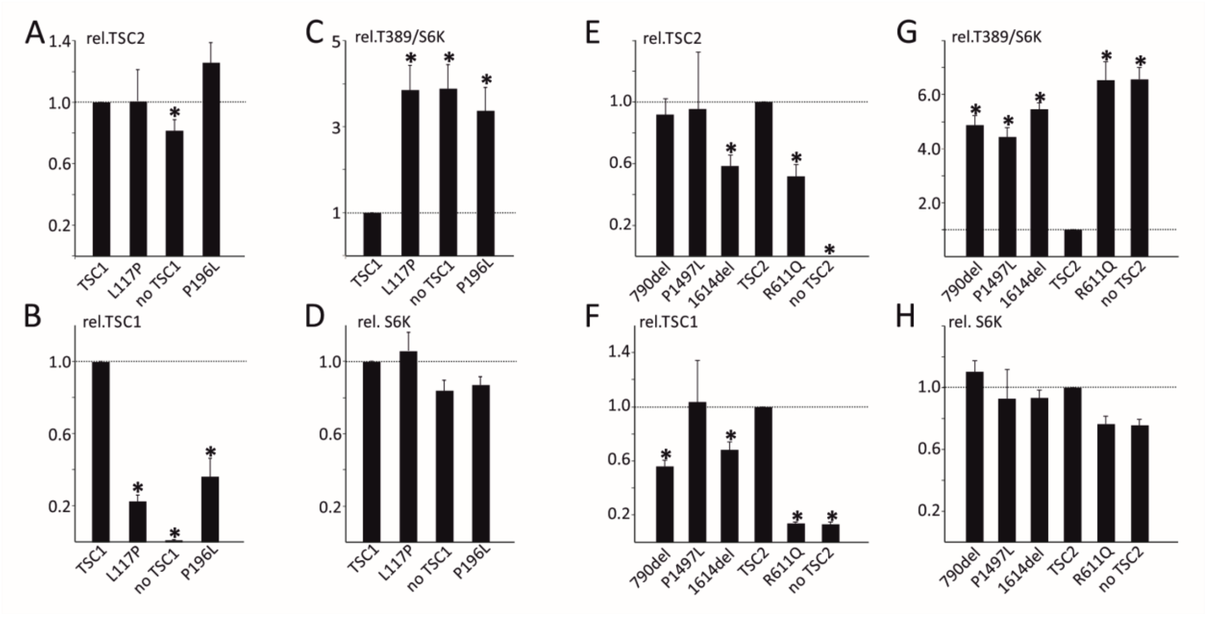
A
rel.TSC2
1.4
1.0
0.6
0.2
*
TSC1
L117P
no TSC1
P196L
B
rel.TSC1
1.0
0.6
0.2
*
*
*
TSC1
L117P
no TSC1
P196L
C
rel.T389/S6K
5
3
1
*
*
*
TSC1
L117P
no TSC1
P196L
D
rel. S6K
1.0
0.6
0.2
TSC1
L117P
no TSC1
P196L
E
rel.TSC2
1.0
0.6
0.2
*
*
*
790del
P1497L
1614del
TSC2
R611Q
no TSC2
F
rel.TSC1
1.4
1.0
0.6
0.2
*
*
*
*
790del
P1497L
1614del
TSC2
R611Q
no TSC2
G
rel.T389/S6K
6.0
4.0
2.0
*
*
*
*
*
790del
P1497L
1614del
TSC2
R611Q
no TSC2
H
rel. S6K
1.0
0.6
0.2
790del
P1497L
1614del
TSC2
R611Q
no TSC2

Figure S3. Detection of large (> 150 bp) deletions using z-scores. The depth of coverage of the subject HaloPlex data was compared to a control cohort across the *TSC1* and *TSC2* loci. The orientation and approximate extent of *TSC1* and *TSC2* are shown on the x-axis; dots represent the z-scores corresponding to the specific HaloPlex probes; red lines indicate the estimated extent of the identified deletions.

(A) Germline NM_000548.3(TSC2):c.1600-182_2546-349del deletion (control DNA).

(B) Post-zygotic NM_000548.3(TSC2): c.(?_-106)-(?_1362-50)del; g.(?_2097990)_(?_2112923)del deletion (subject 2.52).

(C) Post-zygotic NM_000548.3(TSC2): c.(?_-106)_(*102_?)del, p.?, g.(?_2097990)_(2138713_?)del deletion (subject 2.53).

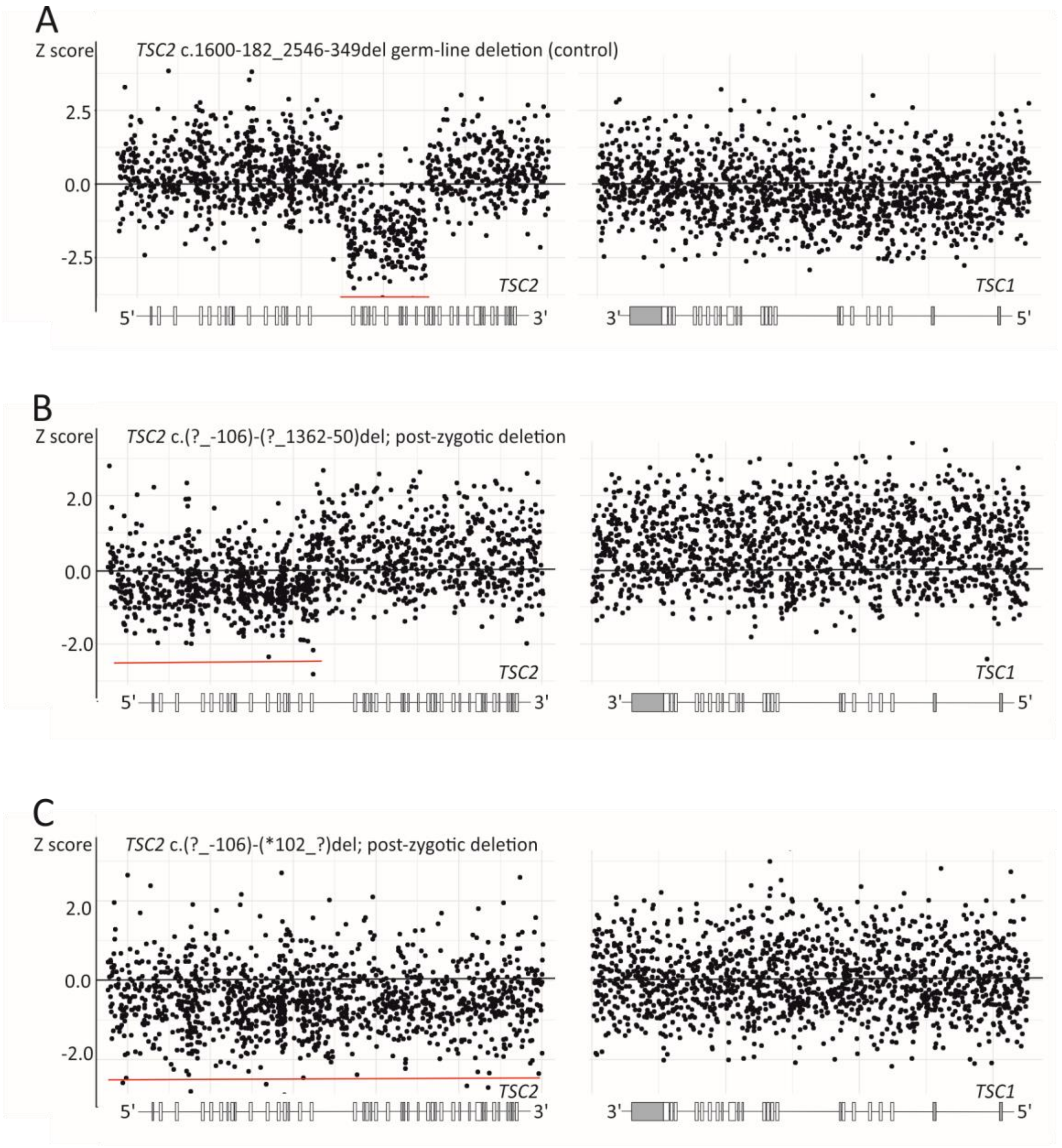
A
Z score
TSC2 c.1600-182_2546-349del germ-line deletion (control)
2.5
0.0
-2.5
TSC2
TSC1
5'
3'
3'
5'
B
Z score
TSC2 c.(?_-106)-(?_1362-50)del; post-zygotic deletion
2.0
0.0
-2.0
TSC2
TSC1
5'
3'
3'
5'
C
Z score
TSC2 c.(?_-106)-(*102_?)del; post-zygotic deletion
2.0
0.0
-2.0
TSC2
TSC1
5'
3'
3'
5'

Figure S4. **Segregation of the NM_000548.3(TSC2):c.1947-23A>G variant.** Pedigree of subjects 1.10 and 1.11 is shown. Individuals reported as affected with TSC are represented by filled circles (females) or squares (males); apparently unaffected individuals by open circles/squares. Genotype of family members for whom genomic DNA was available for testing is indicated.

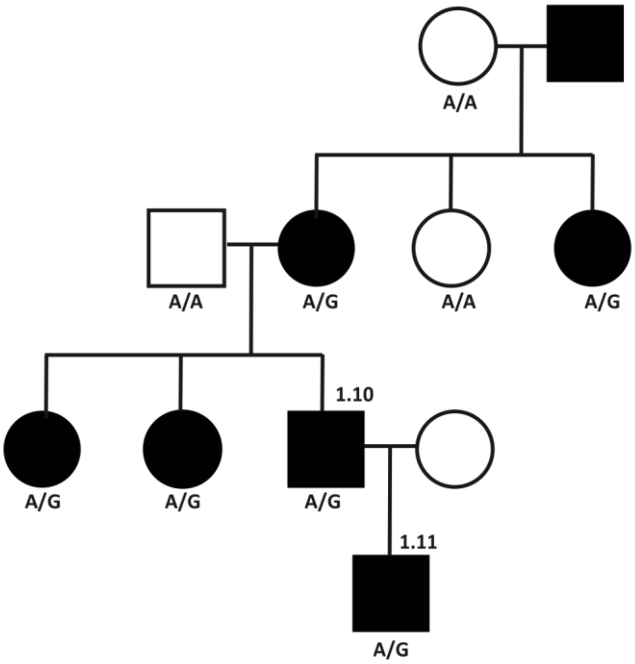

Figure S5. Comparison of the read depth per subject. Scatter/box plot showing the median read depth per subject DNA for individuals with a likely germ-line variant (see main text, Table 1), post-zygotic variant (main text, Table 2) or with no mutation identified (NMI). The median read depths per group were compared using the Wilcoxon rank sum test. We did not obtain evidence that the failure to identify a variant in the NMI group was due to a reduced depth of coverage compared to the other groups. Median read depth was in fact slightly higher in the NMI group, compared to the post-zygotic variant group (Wilcoxon rank sum test *P* = 0.03).

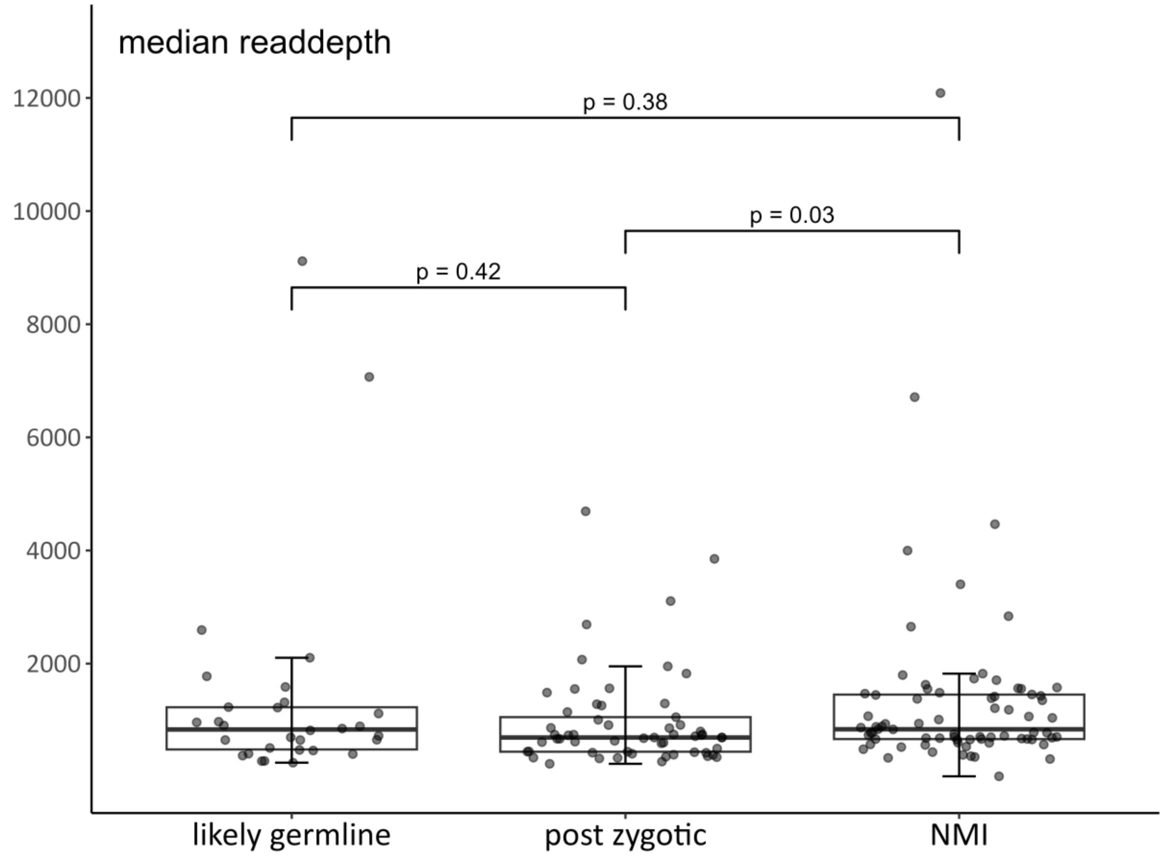

Tables (see SupplTables_30523.docx)

Supplementary Information Table S1: HaloPlex and HaloPlex HS custom capture design characteristics.

Supplementary Information Table S2: HaloPlex and HaloPlex HS custom capture data yield and alignment statistics.

Supplementary Information Table S3: HaloPlex and HaloPlex HS NGS custom capture coverage per subject.

Supplementary Table S4: Clinical features of subjects for whom inactivating, likely germ-line *TSC1* and *TSC2* variants were identified using HaloPlex custom capture NGS.

Supplementary Table S5: Clinical features of subjects for whom inactivating, post-zygotic *TSC1* and *TSC2* variants were identified using HaloPlex custom capture NGS.

Supplementary Table S6: Clinical features of subjects with *TSC1* and *TSC2* variants of uncertain clinical significance (VUS), lesion-specific variants and/or unconfirmed findings identified using HaloPlex custom capture NGS.

Supplementary Information Table S7: Exon trap analysis of *TSC1* variants of uncertain clinical significance that potentially affect pre-mRNA splicing.

Supplementary Information Table S8: Exon trap analysis of *TSC2* variants of uncertain clinical significance that potentially affect pre-mRNA splicing.

Supplementary Information Table S9: Single nucleotide variant (SNV) allele frequencies for copy number variant detection.
