# Supplementary material for: Targeted Genomic Sequencing of TSC1 and TSC2 Reveals Causal Variants in Individuals for Whom Previous Genetic Testing for Tuberous Sclerosis Complex Was Normal
Source: Hum Mutat. 2023 Jul 13;2023:4899372. doi: 10.1155/2023/4899372 (PMC11918493; doi:10.1155/2023/4899372)
Supplement: Supplementary Materials — The Supplementary Information consists of extended methods and the following 5 figures and 9 tables: Figure S1: comparison of the variant allele frequencies (VAF) for selected TSC NMI subjects. Figure S2: Functional assessment of TSC1 and TSC2 variants identified using HaloPlex custom capture NGS. Figure S3. Detection of large (>150 bp) deletions using z-scores. Figure S4: Segregation of the NM_000548.3(TSC2):c.1947-23A>G variant. Figure S5: Comparison of read depths per subject. Supplementary Information Table S1: HaloPlex and HaloPlex HS custom capture design characteristics. Supplementary Information Table S2: HaloPlex and HaloPlex HS custom capture data yield and alignment statistics. Supplementary Information Table S3: HaloPlex and HaloPlex HS NGS custom capture coverage per subject. Supplementary Table S4: Clinical features of TSC NMI subjects for whom inactivating, likely germline TSC1 and TSC2 variants were identified using HaloPlex custom capture NGS. Supplementary Table S5: Clinical features of TSC NMI subjects for whom inactivating, post-zygotic TSC1 and TSC2 variants were identified using HaloPlex custom capture NGS. Supplementary Table S6: Clinical features of TSC NMI subjects with TSC1 and TSC2 variants of uncertain clinical significance (VUS), lesion-specific variants, and/or unconfirmed findings. Supplementary Information Table S7: Exon trap analysis of TSC1 variants of uncertain clinical significance. Supplementary Information Table S8: Exon trap analysis of TSC2 variants of uncertain clinical significance. Supplementary Information Table S9: Single nucleotide variant (SNV) allele frequencies for copy number variant detection. [file 4899372.f1.zip › SupplTables_30523.pdf]

Supplementary Tables

Supplementary Information Table S1: **HaloPlex and HaloPlex HS custom capture design characteristics.** For HaloPlex custom capture of the *TSC1* and *TSC2* loci, 5 different designs, A - E, were employed. For each design the number of amplicons and the extent of the captured regions, according to genome build GRCh37 (hg19) and to the corresponding reference transcripts, are shown. HS: HaloPlex HS; bp: base pairs.

**Design**	***TSC1***					***TSC2***				
	**co-ordinates**		**genomic extent (bp)**	**amplicons**		**co-ordinates**		**genomic extent (bp)**	**amplicons**	
	**GRCh37 (hg19), chr9:**	**NM_000368.4**		**number**	**total sequence (bp)**	**GRCh37 (hg19), chr16**	**NM_000548.3**		**number**	**total sequence (bp)**
**A** (HS)	135760736 _135825020	c.-5234_*10886	64285	2216	338721	2087991 _2139000	c.-10105_*389	51010	1405	273153
**B**	135760736 _135825020	c.-5234_*10886	64285	2337	467654	2087991 _2139000	c.-10105_*389	51010	2588	468773
**C**	135765152 _135824832	c.-5046_*6470	59681	1426	285889	2094729 _21140967	c.-3367_*2356	49229	1193	219072
**D**	135765152 _135824832	c.-5046_*6470	59681	1842	326318	2094729 _21140967	c.-3367_*2356	49229	1587	253364
**E** (HS)	135764736 _135822003	c.-2217_*6886	57267	2230	407835	2095986 _2141492	c.-2110_*2881	45506	2254	336536

Supplementary Information Table S2: **HaloPlex and HaloPlex HS custom capture data yield and alignment statistics.** For HaloPlex custom capture of the *TSC1* and *TSC2* loci, 5 different designs, A - E, were employed. For each design the sequencing protocol, number of samples, sequenced and mapped clusters and the coverage across the captured targeted regions are shown. Overall, between 8 and 67% of the reads were retained after alignment, filtering and UMI deduplication. The mean coverage per amplicon ranged from 16 - 160x per sample, and the overall mean coverage was 70x per amplicon. HS: HaloPlex HS; NGS: Next Generation Sequencing.

**Design**	**NGS protocol**	**samples**	**clusters (min)**	**clusters (max)**	**clusters (mean)**	**mapped (%, min)**	**mapped (%, max)**	**mapped (%, mean)**	**target region coverage**				
									**% coverage (read depth)**				**range**
									**> 20 reads**	**> 100 reads**	**> 300 reads**	**>1000 reads**	
**A** (HS)	MiSeq PE300 V3	15	304088	542658	432436	78.0%	91.1%	84.6%	95.4%	86.3%	57.3%	6.7%	0 - 2453
**B**	MiSeq PE300 V2	48	447240	874018	691387	35.8%	78.8%	54.3%	97.5%	92.1%	79.2%	32.8%	0 - 3859
**C**	HiSeq PE100	48	461075	852798	594141	65.0%	91.6%	81.2%	98.6%	96.2%	91.6%	74.8%	0 - 28287
**D**	HiSeq PE100	89	2278332	71732631	9190992	41.0%	95.6%	86.2%	98.1%	94.9%	87.2%	59.7%	0 - 9057
**E** (HS)	MiSeq PE300 V2	68	1641409	6092131	3819017	89.7%	97.1%	93.9%	97.9%	95.9%	85.8%	29.3%	0 - 3636

Supplementary Information Table S3: HaloPlex and HaloPlex HS NGS custom capture coverage per subject. Median, minimum and maximum read depth per subject DNA and the target region coverage are shown, per design. For subjects for whom multiple genomic DNA samples were analysed, the origin of the DNA is indicated (see the text and Tables 1 - 3 in the main manuscript for details).

Subject	Design	coverage (read depth per nucleotide)			target region covered (%)			
		median	minimum	maximum	read depth > 20	read depth > 100	read depth > 300	read depth > 1000
3.39	A	569	0	3426	96.66	91.88	75.77	18.16
3.25	A	364	0	2218	95.81	88.78	59.60	2.23
3.20 (blood)	A	436	0	2907	95.95	89.70	66.90	7.38
1.18	A	509	0	3120	96.50	91.06	72.49	12.73
2.35	A	407	0	2315	95.87	89.51	65.09	4.11
1.2	A	402	0	2396	95.82	89.55	64.15	4.47
2.17	A	735	0	4697	97.26	92.98	81.84	32.11
2.16	A	353	0	2163	95.29	87.74	57.49	2.15
2.43	A	229	0	1611	94.18	79.96	34.26	0.27
2.8	A	346	0	2156	95.38	87.62	56.95	2.29
2.46 (blood)	A	385	0	2608	95.50	88.54	61.80	4.00
2.46 (fibroblast)	A	426	0	2592	96.24	89.90	66.21	6.36
2.31	A	268	0	1734	94.64	84.26	44.07	0.58
2.52	A	498	0	2654	96.31	90.97	71.63	11.60
3.40	A	525	0	3184	96.62	90.66	72.37	13.98
2.9	A	363	0	2164	95.37	87.97	60.30	2.72
2.19	A	448	0	2758	95.69	89.63	67.89	9.10
1.23	A	249	0	1752	94.64	83.11	39.66	0.26
3.41	A	350	0	2197	95.32	88.36	58.34	2.18
1.10	A	464	0	2879	95.77	89.56	68.57	9.39
1.22	A	277	0	1979	94.89	85.51	45.39	0.79
2.38	A	324	0	1854	95.43	87.00	54.14	1.64
1.4	A	408	0	2422	95.84	89.24	64.86	4.57

2.1	A	443	0	2539	95.64	89.84	67.60	6.95
1.7	A	650	0	3866	96.96	92.45	78.21	23.50
3.42	A	334	0	2751	95.11	87.14	55.56	1.75
2.44	A	434	0	2695	96.42	90.09	66.88	6.97
1.11	A	374	0	2335	95.47	88.40	61.84	3.64
2.54	A	334	0	2029	95.31	87.01	55.32	1.84
1.1	A	474	0	2969	95.78	89.88	69.22	9.74
3.12	A	387	0	2343	95.22	88.74	62.79	3.98
3.12	A	314	0	2145	94.75	84.56	52.50	2.24
1.21	A	973	0	6886	97.70	94.05	84.43	49.00
2.32	A	390	0	2361	96.49	89.12	63.11	4.02
2.23 (blood)	A	314	0	2145	94.75	84.56	52.50	2.24
2.5	B	694	0	3541	97.87	92.02	79.82	30.47
2.18	B	694	0	3536	97.67	91.92	79.21	30.92
3.43	B	603	0	3700	97.22	90.78	74.62	25.49
3.19 (blood)	B	658	0	3324	97.52	92.22	78.25	28.07
3.19 (fibroblasts)	B	704	0	3471	97.89	92.85	80.65	29.90
3.19 (Shagreen)	B	658	0	3676	97.52	91.69	78.38	28.45
3.44	B	1071	0	4977	98.13	94.77	86.45	53.06
2.13	B	671	0	3368	97.64	92.59	79.01	29.49
1.19	B	649	0	3398	97.51	91.40	78.23	28.05
1.8	B	654	0	4262	97.44	90.59	72.97	33.81
1.16	B	820	0	4118	97.83	93.06	82.30	40.15
2.10	B	717	0	4132	97.30	91.70	79.16	33.64
2.12	B	671	0	3607	97.44	91.24	77.27	29.52
3.33	B	891	0	4445	97.97	93.72	83.89	44.26
3.7 (SEGA)	B	699	0	4008	97.64	92.51	80.27	31.16
3.7 (blood)	B	904	0	4563	97.97	93.62	83.79	45.75
3.34	B	706	0	3666	97.56	92.24	79.84	31.39
1.25	B	697	0	4138	97.43	91.94	79.15	32.22
3.20 (angiofibroma)	B	667	0	3532	97.53	92.07	79.03	27.03
2.25	B	860	0	4113	98.05	93.28	83.19	42.23
2.2	B	682	0	3489	97.55	92.09	79.82	29.51
3.16	B	572	0	3967	94.82	87.98	72.69	24.05

2.42	B	620	0	3516	97.58	91.42	76.53	24.26
2.28	B	694	0	4459	97.54	92.36	79.27	31.83
2.8	B	864	0	4180	97.94	93.65	83.81	43.05
2.20	B	1007	0	4814	97.99	94.27	85.45	50.37
2.53	B	748	0	3842	97.76	92.93	79.93	35.83
3.35	B	653	0	3174	97.65	92.45	78.67	25.56
3.11	B	690	0	3471	97.55	92.23	79.46	30.07
2.51	B	424	0	3950	93.66	83.75	63.43	15.04
1.29	B	852	0	5064	97.86	93.85	83.54	42.39
3.36	B	753	0	3946	97.75	92.84	80.82	35.68
2.40	B	603	0	4370	96.92	89.40	70.90	31.34
3.15	B	675	0	3571	97.48	92.12	78.71	29.62
3.37	B	666	0	3363	97.59	91.96	79.05	28.35
3.13	B	487	0	4542	96.57	89.18	68.84	18.61
3.38	B	684	0	3494	96.70	91.47	78.62	29.62
2.37	B	914	0	4284	98.00	93.71	83.82	45.53
1.24	C	7068	0	66904	99.50	98.60	97.19	93.10
3.24	C	1212	0	6825	98.50	95.79	89.47	59.86
3.29	C	27466	0	204347	99.86	99.43	98.79	96.86
2.14	C	3106	0	26674	98.88	96.88	93.65	83.19
2.33	C	4693	0	28206	99.10	97.90	95.77	90.77
1.27	C	1223	0	7681	98.66	95.38	88.73	60.06
3.52	C	4465	0	41423	99.16	98.42	97.00	91.32
2.5	C	3852	0	25888	99.27	98.01	95.75	88.85
1.26	C	9115	0	64082	99.67	98.84	97.72	94.66
3.57	C	3402	0	29332	98.92	97.42	94.51	84.72
1.28	C	2104	0	14726	98.82	96.95	93.12	76.00
2.30	C	2692	0	20924	98.94	96.89	93.18	79.20
3.53	C	1351	0	8411	98.42	94.99	88.31	62.30
3.54	C	12087	0	79175	99.59	98.78	97.54	94.79
2.48	C	1552	0	9605	98.61	95.92	90.74	68.04
3.27	C	1628	0	13581	98.14	94.77	88.69	67.12
3.55	C	1578	0	15665	98.28	95.16	89.09	66.47
3.56	C	1798	0	17750	98.23	95.16	89.74	69.22

1.12	C	1588	0	17364	96.05	92.92	87.72	66.65
1.3	C	2594	0	15505	98.63	96.40	92.80	80.84
2.45	C	1952	0	13987	97.98	95.07	90.11	72.52
3.45	C	1430	0	10259	97.39	93.56	86.95	63.05
2.34	C	1825	0	11160	98.55	95.86	90.91	71.87
3.21	C	1557	0	22552	97.34	92.37	82.98	61.68
3.26	C	1388	0	12060	98.06	94.74	88.32	62.77
3.46	C	1419	0	9533	98.06	94.26	88.24	63.68
3.47	C	2653	0	19416	98.68	96.51	92.49	80.32
3.51	C	2839	0	29136	98.96	97.20	93.88	81.05
3.6	C	3998	0	38408	99.05	97.71	95.10	87.55
3.56	C	6711	0	46176	99.45	98.69	97.06	92.90
2.26	D	713	0	5266	94.48	89.43	74.79	36.60
2.49	D	1145	0	6094	98.46	95.41	88.23	56.48
3.30	D	1708	0	8527	98.53	96.67	92.07	70.91
3.49	D	1011	0	5722	98.09	95.11	86.87	50.50
2.41	D	1259	0	7830	98.35	95.15	88.14	59.84
3.50	D	1486	0	8118	98.50	96.13	90.67	66.67
2.47	D	1283	0	6951	98.03	95.27	88.90	60.83
2.15	D	1295	0	12285	97.47	93.17	85.67	58.62
3.4	D	1446	0	10036	98.01	94.81	88.28	63.79
2.22	D	744	0	5148	97.67	92.25	77.57	37.72
3.58	D	1823	0	8857	98.75	97.06	93.06	74.28
2.39	D	2071	0	11577	98.50	96.84	93.01	74.59
3.59	D	1455	0	8159	98.28	96.11	90.35	64.79
1.13	D	723	0	4329	97.54	93.33	79.80	33.43
1.27	D	1316	0	7725	98.24	95.66	88.70	61.34
3.60	D	1469	0	8057	98.37	95.64	89.86	64.95
3.61	D	1041	0	6296	97.94	94.59	85.43	52.00
2.3	D	444	0	9345	96.23	85.42	62.66	21.70
3.17	D	1552	0	10200	98.24	95.99	90.46	66.93
1.5	D	1231	0	8204	98.26	95.50	88.36	58.97
2.7	D	1488	0	8622	98.63	96.31	90.96	66.26
3.22	D	838	0	5670	97.00	91.73	79.26	42.37

3.31	D	1735	0	14893	98.09	95.15	88.32	66.54
1.17	D	1776	0	14109	98.32	95.78	89.82	68.55
3.48	D	1377	0	7427	97.69	94.87	88.93	63.10
3.32	D	1564	0	9533	98.52	96.30	91.19	68.71
3.68	E	732	0	3383	99.63	99.07	95.23	54.11
3.5 (SEGA FFPE)	E	8	0	22788	17.58	1.32	0.19	0.10
3.5 (blood)	E	778.5	2	3253	99.56	98.40	90.77	32.94
1.15	E	962	2	4112	99.65	98.71	93.64	47.83
2.4	E	613	1	2658	99.47	97.75	85.39	17.92
2.27	E	801	0	3410	99.64	98.46	90.73	35.25
3.3	E	562	2	2474	99.25	97.23	83.20	13.20
1.14 (blood)	E	906	0	4027	99.52	98.76	92.03	43.00
1.14 (SEGA)	E	279	0	1971	98.72	88.64	46.05	2.41
3.10	E	722	1	3048	99.57	98.29	89.48	27.73
3.9	E	841	0	3583	99.66	98.64	92.10	38.66
3.69	E	869	2	3723	99.60	98.61	91.81	40.52
1.6	E	1118	1	4761	99.74	98.74	94.97	57.10
3.70	E	1184	0	4591	99.71	99.03	95.36	60.30
1.9	E	892	1	3644	99.51	98.50	92.35	41.89
2.23 (blood)	E	916	1	3900	99.75	98.81	92.89	44.43
3.2	E	533	0	2154	99.41	97.39	80.24	10.76
3.62	E	1065	0	4354	99.78	99.07	95.23	54.11
3.63	E	935	0	4031	99.70	98.73	93.16	45.52
3.64	E	775	3	3918	99.57	98.41	91.30	32.38
3.65	E	668	0	2485	99.57	97.84	87.29	21.42
3.14	E	683	0	2867	99.47	97.86	87.39	23.86
3.66	E	777	0	2992	99.46	98.40	90.31	33.30
3.1	E	739	1	3220	99.56	98.12	89.44	29.84
2.36	E	635	1	2651	99.46	97.74	86.50	20.32
2.24	E	745	0	3014	99.68	98.17	89.54	28.72
2.11	E	592	1	2268	99.54	97.56	85.75	14.38
3.28	E	783	1	3229	99.66	98.47	90.76	33.46
3.67	E	941	3	4321	99.66	98.97	93.42	45.34
3.23	E	599	2	4121	99.61	98.01	84.87	16.14

2.6	E	338	0	1455	99.28	93.73	57.92	1.26
2.29	E	733	1	2835	99.50	98.47	89.58	27.52
2.21	E	751	2	3353	99.57	98.32	89.87	30.36
3.71	E	705	4	2980	99.55	98.40	89.01	25.28
3.72	E	883	1	3437	99.70	98.95	93.45	41.86

Supplementary Table S4: Clinical features of subjects for whom inactivating, likely germ-line *TSC1* and *TSC2* variants were identified using HaloPlex custom capture NGS. Individuals fulfilling the clinical criteria for definite TSC [5] are indicated with 'TSC'; those fulfilling only criteria for possible TSC are indicated with '?'; individuals for whom clinical information was not available to us are indicated with 'n/a'. The clinical features are reported by organ with the following terms and abbreviations:

N: organ investigated, no abnormality detected; empty cell: organ was not investigated or examined, or the results of the examination or investigation were not available to the study; Tubers: multiple cortical tubers and/or radial migration lines on brain MRI scan; SEN: subependymal nodules on brain MRI scan; SEGA: subependymal giant cell astrocytoma on brain MRI scan or confirmed at surgery; AML: angiomyolipoma; cysts: multiple renal cysts; FA: facial angiofibromas (at least 3); UF: ungual fibromas (at least 2); HMM: hypomelanotic macules (at least 3, >5mm diameter); CSL: confetti skin lesions; FCP: fibrous cephalic plaque; Shagreen: shagreen patch; LAM: lymphangioleiomyomatosis; RM: cardiac rhabdomyoma; RP: retinal phakoma; RAP: retinal achromic patch; GF: gingival fibroma; TAND: TSC-associated neuropsychiatric disorders; VAF: variant allele frequency, refers to the proportion of reads containing the corresponding variant.

Subject	Clinical diagnosis	variant NM_000548.3(TSC2); NM_000368.4(TSC1)	VAF (%)	Brain	Kidney	Skin	Lung	Heart	Eye	Other
1.1	?	*TSC2* c.136A>T, p.(Arg46*)	40%			FA	pulmonary stenosis	RM	RP	
1.2	n/a	*TSC2* c.597_598insTCGT, p.(Gln200Serfs*36)	41%							
1.3, trio	TSC	*TSC2* c.600-124G>A, p.?	47%	tubers, SENs		HMM				
1.4	TSC	*TSC2* c.848+1G>A, p.?	50%	tubers, SENs	cysts	HMM				
1.5, duo	TSC	*TSC2* c.848+281C>T, p.?	50%	tubers, SENs	multiple bilateral AML	FA				affected parent is subject 2.7
1.6	?	*TSC2* c.848+281C>T, p.?	48%			HMM				child of affected parent
1.7	TSC	*TSC2* c.1832G>A, p.(Arg611Gln)	51%	SEN	AML	FA				
1.8	TSC	*TSC2* c.1832G>A, p.(Arg611Gln)	54%	tubers, SEN, SEGA	multiple AML	HMM	N	RM	RP	hepatic micro-AML, TAND
1.9	TSC	*TSC2* c.1947-83G>T, p.?	45%	tubers, SEN		HMM		RM		
1.10, duo	TSC	*TSC2* c.1947-23A>G, r.1947_2002del, p.(Glu650Alafs*34)	33%	tubers, SEN		HMM				4- generation family with TSC; affected parent is subject 1.11
1.11, duo	n/a	*TSC2* c.1947-23A>G, r.1947_2002del, p.(Glu650Alafs*34)	39%							4- generation family with TSC; affected child is subject 1.10
1.12, trio	TSC	*TSC2* c.2221-126C>T, p.?	47%	tubers, SENs		FA, Shagreen, HMM			RAP	
1.13, trio	TSC	*TSC2* c.2369_2371del, p.(Tyr790del)	54%	tubers, SENs,	multiple bilateral AML	HMM			RP	
1.14	TSC	*TSC2* c.2525del, p.(Pro842Hisfs*52)	blood: 48%	SEGA				RM		

			SEGA: 51%							
1.15	TSC	*TSC2* c.2967-460G>A, r.2966ins2967-458_2967-263, p.(Ser989Argfs*82)	51%			HMM, Shagreen				child of affected parent
1.16	n/a	*TSC2* c.3690del, p.(Glu1230Aspfs*25)	43%							
1.17, trio	TSC	*TSC2* c.4006-11G>A, p.?	53%	tubers, SENs	N	FA, HMM, FCP, Shagreen			RAP	enamel pits
1.18	?	*TSC2* c.4490C>G p.(Pro1497Arg)	47%					RM		
1.19	TSC	*TSC2* c.4544_4547del, p.(Asn1515Serfs*60)	49%	tubers, SEN, SEGA	multiple AML	FA, HMM	N	RM	RP	
1.20, duo	TSC	*TSC2* c.4663-3C>G, p.?	44%	SENs	N	HMM		RM		
1.21	n/a	*TSC2* c.4842_4844del p.(Ile1614del)	52%							
1.22	TSC	*TSC2* c.5238_5255del p.(His1746_Arg1751del)	44%		multiple bilateral AML	FA, HMM			RP	GF
1.23	TSC	*TSC1* c.149T>C, p.(Leu50Pro)	51%	tubers		Shagreen		RM		
1.24, trio	TSC	*TSC1* c.363+666T>A, r.363ins68, p.(Met122Aspfs*24)	43%	tubers, SENs	N					
1.25	TSC	*TSC1* c.1431_1434del, p.(Glu478Lysfs*53)	53%	SEGA, SEN, cortical dysplasia		HMM, FA, Shagreen		RM		
1.26, duo	TSC	*TSC1* c.1498C>T, p.(Arg500*)	46%	tubers	N	HMM				
1.27, duo	TSC	*TSC1* c.1498C>T, p.(Arg500*)	51%	tubers, SENs	N	HMM		N		

1.28, trio	TSC	*TSC1* c.1717C>T, (p.Gln573*)	51%	tubers, SEGA		FA, HMM				
1.29	TSC	*TSC1* c.1997+1G>A, p.?	46%	tubers, SENs		FA, Shagreen, UF				

Supplementary Table S5: Clinical features of subjects for whom inactivating, post-zygotic *TSC1* and *TSC2* variants were identified using HaloPlex custom capture NGS. Individuals fulfilling the clinical criteria for definite TSC [5] are indicated with 'TSC'; those fulfilling only criteria for possible TSC are indicated with '?'; individuals for whom clinical information was not available to us are indicated with 'n/a'. The clinical features are reported by organ with the following terms and abbreviations:

N: organ investigated, no abnormality detected; empty cell: organ was not investigated or examined, or the results of the examination or investigation were not available to the study; Tubers: multiple cortical tubers and/or radial migration lines on brain MRI scan; SEN: subependymal nodules on brain MRI scan; SEGA: subependymal giant cell astrocytoma on brain MRI scan or confirmed at surgery; AML: angiomyolipoma; cysts: multiple renal cysts; FA: facial angiofibromas (at least 3); UF: ungual fibromas (at least 2); HMM: hypomelanotic macules (at least 3, >5mm diameter); CSL: confetti skin lesions; FCP: fibrous cephalic plaque; Shagreen: shagreen patch; LAM: lymphangioleiomyomatosis; RM: cardiac rhabdomyoma; RP: retinal phakoma; RAP: retinal achromic patch; GF: gingival fibroma; TAND: TSC-associated neuropsychiatric disorders; VAF: variant allele frequency, refers to the proportion of reads containing the corresponding variant.

Subject	Clinical diagnosis	variant NM_000548.3(TSC2); NM_000368.4(TSC1)	VAF	Brain	Kidney	Skin	Lung	Heart	Eye	Other
2.1	TSC	*TSC2* c.139_140del, p.(Glu47Thrfs*19)	12%	tubers, SENs	cysts	FA, HMM, Shagreen				
2.2	n/a	*TSC2* c.139-2A>G, p.?	7%							
2.3	TSC	*TSC2* c.268C>T, p.(Gln90*)	7%	SEGA	multiple bilateral AML	FA	LAM			
2.4	?	*TSC2* c.268C>T, p.(Gln90*)	5%		multiple AML					
2.5	n/a	*TSC2* c.299del, p.(Ala100Glyfs*6)	2%							
2.6	TSC	*TSC2* c.352dup, p.(Val118Glyfs*8)	2%	SENs				RM		
2.7, duo	TSC	*TSC2* c.848+281C>T, p.?	4%	tubers	multiple bilateral AML	FA, UF				affected child is subject 1.5
2.8	TSC	*TSC2* c.848+281C>T, p.?	29%		AML	FA, UF				GF
2.9	TSC	*TSC2* c.976-15G>A, p.?	4%	tubers	AML	FA				GF
2.10	n/a	*TSC2* c.990_1005del, p.(Asn331Metfs*27)	11%							
2.11	TSC	*TSC2* c.1120-28_1120-10del, p.?	7%	tubers, SENs	N	HMM	N	N	N	
2.12	TSC	*TSC2* c.1210C>T, p.(Gln404*)	11%	cortical dysplasia, SEN	cysts, AML	HMM, FA, Shagreen		RM	RP	
2.13	TSC	*TSC2* c.1221C>A, p.(Tyr407*)	3%	N	multiple AML	FA	N	N	N	TAND, psoriasis
2.14, trio	TSC	*TSC2* c.1258-1G>A, p.?	8%		multiple bilateral AML	FA, HMM, FCP			N	

2.15,<br>trio	TSC	*TSC2* c.1361+1G>A, p.?	3%	tubers	N	FA	LAM			
2.16	TSC	*TSC2* c.1372C>T<br>p.(Arg458*)	4%	tubers, SENs		FA, HMM				
2.17	n/a	*TSC2* c.1492G>T<br>p.(Glu498*)	23%							
2.18	TSC	*TSC2* c.1636del,<br>p.(Glu546Lysfs*15)	2%	cortical<br>dysplasia	AML,<br>cysts	HMM, FA				
2.19	TSC	*TSC2* c.1831C>T,<br>p.(Arg611Trp)	4%	tubers, SEN	AML	FA, FCP	LAM			
2.20	TSC	*TSC2* c.1831C>T,<br>p.(Arg611Trp)	3%	tubers	AML	HMM, FA			RAP	
2.21	TSC	*TSC2* c.1832G>A,<br>p.(Arg611Gln)	1%	SEN	multiple,<br>bilateral<br>AML					
2.22,<br>trio	TSC	*TSC2* c.1852del<br>p.(Leu618Cysfs*80)	19%		multiple<br>bilateral<br>AML,<br>cysts	FA		RM		
2.23	TSC	*TSC2* c.2108G>A,<br>p.(Trp703*)	blood: 8%<br>blood: 7%	SEN		FA				
2.24	TSC	*TSC2*<br>c.2119_2120ins[2098_21<br>19;GTCT],<br>p.(Lys707Argfs*3)	2%	tubers, SENs	multiple<br>bilateral<br>AML	FA, HMM, UF	N	N	N	
2.25	TSC	*TSC2* c.2251C>T<br>p.(Arg751*)	3%			FA, UF				
2.26,<br>trio	TSC	*TSC2* c.2590C>T,<br>p.(Gln864*)	2%	tubers, SENs	multiple<br>bilateral<br>AML	FA, HMM, FCP				
2.27	?	*TSC2* c.2590_2593dup,<br>p.(Tyr865fs*19)	1%		AML					
2.28	TSC	*TSC2* c.2687G>A,<br>p.(Trp896*)	2%		AML	FCP, HMM,<br>UF				enamel pits

2.29	?	*TSC2* c.2713C>T, p.(Arg905Trp)	1%	SEN						
2.30, trio	TSC	*TSC2* c.2742G>A, p.(Lys914=)	13%	SENs	multiple bilateral AML	FA			RP	
2.31	TSC	*TSC2* c.2838-122G>A, r.2837ins120fs, p.(Ser949Argins4*)	36%	SEN		HMM			RP	
2.32	TSC	*TSC2* c.2838-122G>A, r.2837ins120fs, p.(Ser949Argins4*)	31%	tubers, SENs				RM		
2.33, duo	TSC	*TSC2* c.2838-122G>A, r.2837ins120fs, p.(Ser949Argins4*)	15%	tubers, SENs	multiple bilateral AML	FA				
2.34, trio	TSC	*TSC2* c.2838-122G>A, r.2837ins120fs, p.(Ser949Argins4*)	15%	tubers, SENs	N	FA, HMM		RM		
2.35	?	*TSC2* c.2838-122G>A, r.2837ins120fs, p.(Ser949Argins4*)	20%			HMM, Shagreen, UF				
2.36	TSC	*TSC2* c.3094C>T, p.(Arg1032*)	2%	N	multiple bilateral AML, cysts	FA	N	N	N	single HMM
2.37	TSC	*TSC2* c.3412C>T p.(Arg1138*)	1%		AML	FA, HMM				
2.38	TSC	*TSC2* c.3412C>T p.(Arg1138*)	16%	SENs		FA, HMM				
2.39, trio	TSC	*TSC2* c.3520del, p.(Arg1174Glyfs*17)	3%	SENs	multiple bilateral AML	FA		N	RP	
2.40	TSC	*TSC2* c.3696dup, p.(Asn1233*)	3%	tubers	AML	FA, HMM				

2.41, trio	TSC	*TSC2* c.4351dup p.(Arg1451Profs*73)	7%	tubers	multiple bilateral AML	FA				
2.42	?	*TSC2* c.4488_4491C[6], p.(Ser1498Profs*79)	11%			HMM, FA				
2.43	TSC	*TSC2* c.4490C>T, p.(Pro1497Leu)	13%	tubers, SENs		HMM				
2.44	TSC	*TSC2* c.4537G>T, p.(Glu1513*)	13%	SEN		HMM				
2.45, trio	TSC	*TSC2* c.4959C>A, p.(Ser1653=)	25%	tubers, SENs	N	FA, HMM, Shagreen				
2.46	TSC	*TSC2* c.5024C>T p.(Pro1675Leu)	18%	tubers, SENs	AML	FA				
2.47, trio	TSC	*TSC2* c.5069-2A>G, p.?	3%	tubers, SENs, SEGA	multiple bilateral AML	FA, HMM, Shagreen		N		GF
2.48, trio	TSC	*TSC2* c.5183_5184insGCCG, p.(Ser1728Argfs*48)	20%	tubers						
2.49, trio	TSC	*TSC2* c.5227C>T, p.(Arg1743Trp)	12%	tubers	multiple bilateral AML	FA, HMM, Shagreen				
2.50, trio	TSC	*TSC2* c.5228G>A, p.(Arg1743Gln)	7%	SENs	cysts	HMM				
2.51	TSC	*TSC2* c.5228G>A, p.(Arg1743Gln)	17%	tubers, SEN	AML	FA, FCP				
2.52, duo	TSC	*TSC2* c.(?_-106)_(?_1362-50)del, p.?	~15%	tubers, SENs	AML			RM		
2.53	TSC	*TSC2* c.(?_-106)_(*102_?)del, p.?	~10%	tubers, SENs, SEGA	AML	FA				
2.54	TSC	*TSC1* c.587C>T p.(Pro196Leu)	17%	tubers, SENs	cysts	FA, HMM, Shagreen, UF			RAP	

Supplementary Table S6: Clinical features of subjects with *TSC1* and *TSC2* variants of uncertain clinical significance (VUS), lesion-specific variants and/or unconfirmed findings identified using HaloPlex custom capture NGS. Individuals fulfilling the clinical criteria for definite TSC [5] are indicated with 'TSC'; those fulfilling only criteria for possible TSC are indicated with '?'. The clinical features are reported by organ with the following terms and abbreviations: N: organ investigated, no abnormality detected; empty cell: organ was not investigated or examined, or the results of the examination or investigation were not available to the study; Tubers: multiple cortical tubers and/or radial migration lines on brain MRI scan; SEN: subependymal nodules on brain MRI scan; SEGA: subependymal giant cell astrocytoma on brain MRI scan or confirmed at surgery; AML: angiomyolipoma; cysts: multiple renal cysts; FA: facial angiofibromas (at least 3); UF: ungual fibromas (at least 2); HMM: hypomelanotic macules (at least 3, >5mm diameter); CSL: confetti skin lesions; FCP: fibrous cephalic plaque; Shagreen: shagreen patch; LAM: lymphangioleiomyomatosis; RM: cardiac rhabdomyoma; RP: retinal phakoma; RAP: retinal achromic patch; GF: gingival fibroma; TAND: TSC-associated neuropsychiatric disorders; ADPKD: autosomal dominant polycystic kidney disease (molecular confirmation); VAF: variant allele frequency, refers to the proportion of reads containing the corresponding variant.

**Subject**	**Diagnosis**	**variant** NM_000548.3(TSC2); NM_000368.4(TSC1)	**VAF**	**Brain**	**Kidney**	**Skin**	**Lung**	**Heart**	**Eye**	**Other**
3.1	TSC	*TSC2* c.226-1222G>T, p.?	43%	tubers	N	FA, HMM, CSL	N	N	N	
3.2	?	*TSC2* c.337-183G>A, p.?	41%			FA				
		TSC2 c.3397+230C>T, p.?	48%							
3.3	?	*TSC2* c.482-400T>C, p.?	55%	SENs		HMM				
		TSC2 c.1716+284C>G, p.?	6%							
3.4, trio	TSC	*TSC2* c.529dup p.(Leu177Profs*12) (unconfirmed)	2%	tubers, SENs	multiple bilateral AML	FA	LAM			enamel pits
3.5	TSC	*TSC2* c.599+4A>G, p.? (unconfirmed)	blood: 0% SEGA (FFPE): 30%	SEGA	AML	FA				
3.6, trio	TSC	*TSC2* c.1600-3_1656del, p.? (unconfirmed)	0.3%							
3.7	?	*TSC2* c.4375C>T p.(Arg1459*)	blood: 0% SEGA: 53%	SEGA						
3.9	?	*TSC2* c.1717-785_1717-784del	49%		AML		LAM			
3.10	?	*TSC2* c.2859del, p.(Lys954Asnfs*4)	1%		AML	HMM				
3.11	?	*TSC2* c.4006-118G>A, p.?	29%	tubers, SENs		HMM				
3.12	TSC	*TSC2* c.5161-32A>C, p.?	46%			FA, FCP, HMM, Shagreen, UF				
3.13	?	*TSC1* c.363+334C>T, p.?	42%	tubers						
3.14	TSC	*TSC1* c.363+633_363+634delinsTT, p.?	44%	tubers	N	FA, HMM, FCP	N	N	N	

3.15	TSC	*TSC1* c.1264-728T>G, p.?	51%	tubers, SENs, SEGA						
3.16	?	*TSC1* c.1997+17C>G, p.?	37%		AML, cysts	HMM				ADPKD
3.17	?	*TSC1* c.2392-110G>C, p.?	42%	N	multiple bilateral AML	N			N	
3.19	?	*TSC2* c.5024C>T p.(Pro1675Leu), chr16:2137898C>T	blood: 0% fibroblasts: 0% shagreen patch: 2%	N	N	Shagreen	N	N	N	
3.20	TSC	*TSC2* c.*141G>T, p.?<br>*TSC2* c.1331del, p.(Asn444Thrfs*5)	blood: 20% angiofibroma: 23%<br>blood: 0% angiofibroma: 3%	cortical dysplasia	AML	HMM, FA, Shagreen			RP	
3.21, trio	TSC	*TSC2* c.5200G>T p.(Asp1734Tyr) (unconfirmed)	3%	tubers, SENs	cysts	N		RM		
3.22	TSC	*TSC1* c.1439-57G>T, p.? (unconfirmed)	1%	tubers	multiple bilateral AML	FA, UF				
3.23	?	NMI		N	N	HMM	N	N	N	N
3.24	?	NMI		N	multiple bilateral AML	N			N	
3.25	TSC	NMI		tubers, SENs, SEGA		FA, HMM				
3.26	TSC	NMI		tubers, SENs	cysts	HMM		RM		
3.27	TSC	NMI		tubers						
3.28	TSC	NMI		tuber	multiple bilateral AML, cysts	FA, HMM	N	N	N	

3.29	TSC	NMI		tubers, SENs	cysts	HMM		RM	RP	
3.30	TSC	NMI		tubers, SENs	multiple bilateral AML	CSL				
3.31	TSC	NMI		tubers, SENs	multiple bilateral AML	N				
3.32	?	NMI		SENs	N	N		N	N	
3.33	TSC	NMI		SENs, cortical dysplasia	AML	FA, Shagreen				
3.34	TSC	NMI		SENs, cortical dysplasia, SEGA	AML, cysts	HMM, FA, Shagreen			RP	
3.35	TSC	NMI		tubers	AML	FCP, HMM, UF				
3.36	TSC	NMI		SEN		Shagreen				
3.37	?	NMI			AML				RAP	
3.38	?	NMI					LAM			
3.39	TSC	NMI		tubers		HMM		RM		
3.40	TSC	NMI				FA, FCP, HMM, Shagreen, UF				GF
3.41	TSC	NMI			AML	HMM		RM		
3.42	TSC	NMI		tubers	N	FA, Shagreen		N	N	TAND
3.43	TSC	NMI		tubers, SENs		HMM, FA				
3.44	TSC	NMI		tubers, SENs	cysts			RM		
3.45	TSC	NMI		tubers	multiple bilateral AML	FA				

3.46	TSC	NMI		tubers, SENs	multiple bilateral AML	FA, HMM				
3.47	TSC	NMI		tubers, SENs, SEGA	multiple bilateral AML	N				
3.48	TSC	NMI		tubers, SENs		HMM				
3.49	TSC	NMI		tubers, SENs	N	N		RM		
3.50	TSC	NMI		SENs	multiple bilateral AML	HMM				
3.51	TSC	NMI		tubers	multiple bilateral AML	FA	N			
3.52	TSC	NMI		tubers, SENs	N	FA, Shagreen				
3.53	?	NMI		unspecified abnormalities on brain MRI	N	N		N	N	
3.54	TSC	NMI		tubers, SENs	multiple bilateral AML, cysts	FA, UF			RP, RAP	
3.55	TSC	NMI		SENs	multiple bilateral AML	FA			N	
3.56	TSC	NMI		tubers, SENs	multiple bilateral AML	FA, UF, HMM		RM	N	
3.57	?	NMI		N	multiple bilateral AML	N		N	N	
3.58	TSC	NMI		tuber, SENs	multiple bilateral AML	HMM				

3.59	?	NMI		N	multiple bilateral AML	N			N	
3.60	TSC	NMI		tubers, SENs	N	N		RM	RAP	
3.61	TSC	NMI		tubers, SENs	multiple bilateral AML	FA				GF
3.62	?	NMI			AML	HMM				
3.63	TSC	NMI		tubers	N	FA	N	N	N	
3.64	TSC	NMI		SENs, SEGA	multiple AML	FA, HMM, FCP	N	N	N	
3.65	TSC	NMI		tubers, SENs	N	FA, Shagreen, UF	N	N	N	
3.66	TSC	NMI		tubers	multiple bilateral AML	FA, FCP	N			
3.67	TSC	NMI		tubers	N	FA, HMM, UF	N	N	N	
3.68	TSC	NMI		cortical dysplasia, SEN		HMM		RM	RP	
3.69	TSC	NMI		tuber, SEN						
3.70	?	NMI						RM		
3.71	?	NMI			multiple bilateral AML					
3.72	?	NMI			AML	FA				

Supplementary Information Table S7: Exon trap analysis of *TSC1* variants of uncertain clinical significance that potentially affect pre-mRNA splicing. *TSC1* variants suspected of affecting pre-mRNA splicing and identified in individuals with a definite or possible clinical diagnosis of TSC were cloned into the pSPL3 exon trap vector, as indicated, and compared to the corresponding wild-type exon trap construct using RT-PCT and Sanger sequencing.

variant: NG_012386.1, NM_000368.4(TSC1)	pSPL3 construct	Observation
c.363+334C>T	exon 5<br>c.211-126_363+546	no effect on splicing
c.664-15A>G	exon 8<br>c.664-132_737+193	r.664_737del, p.(Pro222Valfs*8); r.663-664ins664-14_664-1, p.(Met223Glnfs*4)
c.738-20A>G	exon 9<br>c.738-202_913+118	no effect on splicing
c.1264-728T>G	exon 13<br>c.1264-776_1333+124	no effect on splicing
c.1997+17C>G	exon 15<br>c.1439-170_1997+120	no effect on splicing
c.1572C>G + c.1997+17C>G	exon 15<br>c.1439-170_1997+120	no effect on splicing
c.2042-5A>G	exon 17<br>c.2042-147_2208+113	r.2041ins2042-4_2042-1, p.(Gly681Valfs*8)
c.2209-12G>A	exon 18<br>c.2209-243_2391+102	r.2208ins2209-10_2209-1, p.(Lys737Serfs*20)
c.2392-110G>C	exon 19<br>c.2392-186_2502+115	no effect on splicing
c.2503-11T>A	exon 20<br>c.2503-92_2625+492	no effect on splicing
c.2625G>A	exon 20<br>c.2503-92_2625+492	r.2503_2625del, p.(Leu835_Lys875del)
c.2625+367A>G	exon 20<br>c.2503-92_2625+492	r.2625ins2625+275_2625+362, [r.2625ins88] p.(Lys875ins29*)

Supplementary Information Table S8: Exon trap analysis of *TSC2* variants of uncertain clinical significance that potentially affect pre-mRNA splicing. *TSC2* variants suspected of affecting pre-mRNA splicing and identified in individuals with a definite or possible clinical diagnosis of TSC were cloned into the pSPL3 exon trap vector, as indicated, and compared to the corresponding wild-type exon trap construct using RT-PCT and Sanger sequencing.

**variant: NG_005895.1, NM_000548.3(TSC2)**	**pSPL3 construct**	**Observations**
c.-29-4A>C	exon 2<br>c.-29-111_138+119	(50%): r.-29_138del p? start loss; alternative ATG in exon 3: p.(Met1_Ser49del)
c.138+5G>A	exon 2<br>c.-29-111_138+119	r.-29_138del p? start loss; alternative ATG in exon 3: p.(Met1_Ser49del)
c.226-6T>G	exon 4<br>c.226-127_336+97	r.226_336del p.(His76_Gln112del)
c.337-174G>A	exons 4 + 5<br>c.226-127_481+108	no effect on splicing.
c.585C>T	exons 6 +7<br>c.482-208_648+151	r.482_599delins[c.600-216_600-1], p.Ala161Glu*47;<br>r.482_599del, p.Ala161Glyfs*2
c.585C>A	exons 6 +7<br>c.482-208_648+151	r.482_599delins[c.600-216_600-1], p.Ala161Glu*47;<br>r.482_599del, p.Ala161Glyfs*2
c.599+3A>G	exons 6 +7<br>c.482-208_648+151	r.482_599delins[c.600-216_600-1], p.Ala161Glu*47;<br>r.482_648delins[c.600-216_600-90], p.(Ala161Glufs*81)
c.599+4A>G	exons 6 +7<br>c.482-208_648+151	r.482_599delins[c.600-216_600-1], p.Ala161Glu*47;<br>r.482_648delins[c.600-216_600-90], p.(Ala161Glufs*81)
c.599+5G>A	exons 6 +7<br>c.482-208_648+151	r.482_599delins[c.600-216_600-1], p.Ala161Glu*47;<br>r.482_648delins[c.600-216_600-90], p.(Ala161Glufs*81)
c.600-145C>T intron 6	exons 6 +7<br>c.482-208_648+151	r.482_599delins[c.600-216_600-147],<br>p.(Ala161_Gln200delins24)
c.1033_1035delinsTTA	exon 11<br>c.976-124_1119+121	r.976_1119del, p.(Ala326_Gln373del)
c.1361+146C>T	exons 13 + 14<br>c.1258-96_1443+227	no effect on splicing
c.1624C>G, p.(Pro542Ala)	exon 16	no effect on splicing

	c.1600-77_1716+181	
c.1716+5G>A	exon 16<br>c.1600-77_1716+181	r.1600_1716del p.(Val534_Gln572del)
c.1716+5G>C	exon 16<br>c.1600-77_1716+181	r.1600_1716del p.(Val534_Gln572del)
c.2098-7_2098-5delCTC	exon 20<br>c.2098-121_2220+124	r.2098_2220del, p.(Glu700_Met740del)
c.2220+3A>C	exon 20<br>c.2098-121_2220+124	r.2098_2220del, p.(Glu700_Met740del)
c.2220+96G>A	exon 20<br>c.2098-121_2220+124	no effect on splicing
c.2355+392C>G	exon 22<br>c.2355+167_2545+230	no effect on splicing
c.2743-4_2743dup5	exon 25<br>c.2743_105_2837+89	r.2742insGCCAG, p.(Lys914fs34*?);<br>r.2743_2837del, p.(Gly915Serfs*13)
c.2743-3C>A	exon 25<br>c.2743_105_2837+89	r.2743_2837del, p.(Gly915Serfs*13)
c.2838-122G>A	exon 26<br>c.2838-249_2966+98	r.2837ins120, p.(Ser946Argins4*)
c.3397+5G>T	exons 27 - 29<br>c.2967-73_3397+86	r.3285_3397del, p.(Ser1095Argfs*35)
c.4590G>A, p.=	exons 35 + 36<br>c.4493+46_4662+138	r.4570_4662del, p.(Gln1525_Ser1555del)
c.4662G>C, p.(Gln1554His)	exons 35 + 36<br>c.4493+46_4662+138	r.4570_4662del, p.(Gln1525_Ser1555del)
c.4959C>A, p.=	exon 38<br>c.4850-95_4989+113	r.4961_4989del, p.Glu1655Profs*41
c.4990-6G>A intron 38	exons 39 - 41<br>c.4990-124_5266	r.4989insGCAG, p.(Lys1663fs*9)
c.5161-32A>C	exons 39 - 41<br>c.4990-124_5266	no effect on splicing
c.5161-1G>A	exons 39 - 41<br>c.4990-124_5225	r.5161_5173del, p.(Met1721_Gln1724del)

Supplementary Information Table S9: Single nucleotide variant (SNV) allele frequencies for copy number variant detection. Comparison of variant allele frequencies (VAFs) for informative SNVs mapping to the TSC2 locus (GRCh37 (hg19) chr16:2095986_2141492) for subject 2.52; NM_000548.3(TSC2):c.(?_-106)_(?_1362-50)del; chr16:(?_2097990)_(?_2112923)del post-zygotic deletion), their unaffected sibling and subject 2.53; NM_000548.3(TSC2):c.(?_-106)_(*102_?))del; chr16:(?_2097990)_(2138713_?)del post-zygotic deletion). SNVs with a VAF < 40% are indicated in bold. Shaded cells indicate the extent of the deleted region in subjects 2.52 and 2.53.

GRCh37 (hg19) chr16:g.	VAF (% read counts)		
	unaffected sibling of 2.52	subject 2.52	subject 2.53
2099232A>G	58	42	
2102486T>A	51	**36**	
2102611A>G	54	**28**	
2105055A>G	53	**37**	
2109048insTT	47	**38**	
2110571C>G	56	**39**	
2111230A>G	48	40	
2111779T>C	49	**38**	
2114644T>G	55	54	
2115819A>G	52	51	**29**
2121001A>C	48	47	
2130781A>G			46
2132282A>G			59
2134221C>T	50	56	
2138201delAGTG	**36**	**26**	
